# Electron Acceptor-Driven Solid Electrolyte Interphases with Elevated LiF Content for 4.7 V Lithium Metal Batteries

**DOI:** 10.1007/s40820-025-01663-x

**Published:** 2025-02-24

**Authors:** Yongbiao Mu, Zifan Liao, Youqi Chu, Qing Zhang, Lingfeng Zou, Lin Yang, Yitian Feng, Haixiang Ren, Meisheng Han, Lin Zeng

**Affiliations:** 1 Shenzhen Key Laboratory of Advanced Energy Storage, Department of Mechanical and Energy Engineering, Southern University of Science and Technology, Shenzhen, 518055 People’s Republic of China; 2 SUSTech Energy Institute for Carbon Neutrality, Southern University of Science and Technology, Shenzhen, 518055 People’s Republic of China

**Keywords:** Lithium metal batteries, High-voltage cathodes, Electron acceptor, Dendrite formation, Dual interfaces

## Abstract

High-voltage lithium (Li) metal batteries (LMBs) face substantial challenges, including Li dendrite growth and instability in high-voltage cathodes such as LiNi_0.8_Mn_0.1_Co_0.1_O_2_ (NCM811), which impede their practical applications and long-term stability. To address these challenges, tris(pentafluorophenyl)borane additive as an electron acceptor is introduced into an ethyl methyl carbonate/fluoroethylene carbonate-based electrolyte. This approach effectively engineers robust dual interfaces on the Li metal anode and the NCM811 cathode, thereby mitigating dendritic growth of Li and enhancing the stability of the cathode. This additive-driven strategy enables LMBs to operate at ultra-high voltages up to 4.7 V. Consequently, Li||Cu cells achieve a coulombic efficiency of 98.96%, and Li||Li symmetric cells extend their cycle life to an impressive 4000 h. Li||NCM811 full cells maintain a high capacity retention of 87.8% after 100 cycles at 4.7 V. Additionally, Li||LNMO full cells exhibit exceptional rate capability, delivering 132.2 mAh g^−1^ at 10 C and retaining 95.0% capacity after 250 cycles at 1 C and 5 V. As a result, NCM811||graphite pouch cells maintain a 93.4% capacity retention after 1100 cycles at 1 C. These findings underscore the efficacy of additive engineering in addressing Li dendrite formation and instability of cathode under high voltage, thereby paving the road for durable, high-performance LMBs.

**Supplementary Information:**

The online version contains supplementary material available at 10.1007/s40820-025-01663-x.

## Highlights

- A tris(pentafluorophenyl)borane additive as an electron acceptor is incorporated into an ethyl methyl carbonate/fluoroethylene carbonate/lithium nitrate electrolyte.
- This approach effectively engineers durable dual interfaces on both lithium metal anode and LiNi_0.8_Mn_0.1_Co_0.1_O_2_ (NCM811) cathode, which mitigates dendritic growth and enhances cathode stability.
- The additive-driven strategy enables lithium metal batteries to operate at ultra-high voltage up to 4.7 V and high mass loading of 14.0 mg cm^−2^ for NCM811 cathode, thus resulting in exceptional cycling performance.

**Supplementary Information:**

The online version contains supplementary material available at 10.1007/s40820-025-01663-x.

## Graphical abstract

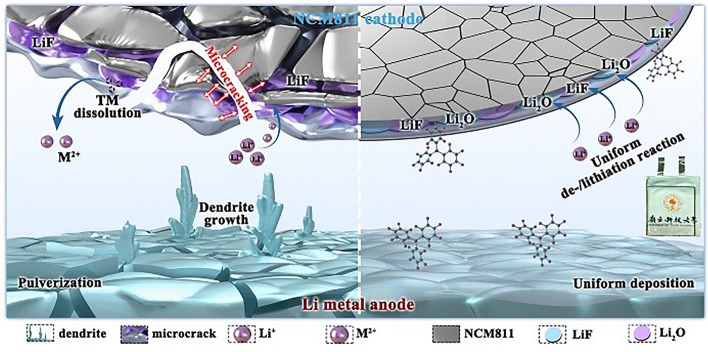

## Introduction

Lithium (Li) metal batteries (LMBs) featuring high-voltage cathodes have garnered considerable attention due to their potential to significantly enhance energy density and performance [1–3]. Among these, nickel-rich cathodes (e.g., LiNi_0.8_Co_0.1_Mn_0.1_O_2_ (NCM811) [4–6]) and high-voltage spinel materials (e.g., LiNi_0.5_Mn_1.5_O_4_ (LNMO) [7, 8]) are promising candidates for achieving high operating voltages above 4.5 and 5 V, respectively. High-voltage cathodes provide several advantages, including enhanced energy density and improved power output, which are crucial for extending the driving range and reducing the charging time of electric vehicles [9–11]. However, these cathodes also present significant challenges, particularly concerning the stability of the electrolyte and the formation of a robust solid electrolyte interphase (SEI) [12]. NCM811 and LNMO cathode materials face issues such as solvent decomposition, transition metal dissolution, and unstable cathode-electrolyte interphase (CEI) layers at high voltages, making the development of suitable high-voltage electrolytes crucial. The use of new high-oxidation-resistant solvents, such as sulfone-based electrolytes and fluorine-containing solvents (e.g., FEC) [13], can effectively enhance the electrolyte's tolerance to high voltage and form stable CEI layers in the battery. Common additives include phosphorus-based (e.g., dimethyl methylphosphonate) [14], boron-based (e.g., tri-n-butyl borate) [15], silicon-based (e.g., tris(pentafluorophenyl)silane) [16, 17], sulfur-based (e.g., *p*-toluenesulfonyl isocyanate) [18], among others. These additives help improve cycling stability and high-voltage performance by forming stable films on the electrode surface, inhibiting solvent decomposition, and removing by-products (e.g., HF and H_2_O), thus enhancing the overall performance of the Li metal batteries [19]. Additionally, the Li metal anodes, despite their high theoretical capacity and low electrochemical potential, pose significant challenges, such as dendrite formation during repeated cycling, which can cause short circuits and potential safety hazards [20, 21]. The formation of Li dendrites, coupled with the high reactivity of Li metal with organic electrolytes, results in the continuous consumption of active lithium and electrolytes, further compromising the battery's performance and lifespan. Therefore, addressing these challenges is crucial for realizing the full potential of LMBs with high-voltage cathodes [9, 22, 23].

Recent research efforts have concentrated on developing high-voltage electrolytes for NCM811 and LNMO materials that can operate stably at voltages exceeding 4.5 V [24–26]. These strategies are generally categorized into two main approaches: the incorporation of additives and the optimization of solvent and lithium salt formulations. Additives play a crucial role in stabilizing high-voltage electrolytes by forming a robust and protective solid electrolyte interface layer, which can reduce decomposition of electrolytes and enhance the overall stability of the batteries. Various additives have been investigated for their effectiveness in high-voltage environments. Film-forming additives, such as FEC [27, 28] and vinylene carbonate (VC) [29, 30] form stable SEI layers on the anode, mitigate lithium dendrite formation, and enhance electrolyte stability. Oxidation-resistant additives, such as lithium bis(fluorosulfonyl)imide (LiFSI) and lithium difluoro(oxalato)borate (LiDFOB) [31–33], assist in forming a stable CEI that resists oxidation, a critical factor for high-voltage cathodes like NCM811 and LNMO. Lithium nitrate (LiNO_3_) [34–36] is widely used due to its ability to form a stable and uniform SEI layer on the Li anodes, thereby preventing dendrite growth and significantly improving cycling stability and efficiency. The choice of solvents and lithium salts significantly influences the performance and stability of high-voltage electrolytes.

Recent research has focused on identifying optimal combinations to enhance the electrochemical stability window and improve ion transport properties. Solvents such as ethyl methyl carbonate (EMC) and dimethyl carbonate (DMC) are commonly combined with more stable solvents like FEC to enhance the stability of electrolytes under high voltage, thereby balancing the trade-offs between ionic conductivity, viscosity, and electrochemical stability. High-voltage solvents with superior oxidative stability, such as sulfolane and dioxolane [37], have been investigated for their potential to enhance electrolyte stability at high voltages by resisting oxidative conditions at the cathode interface, thereby reducing electrolyte decomposition and extending cycle life. While lithium hexafluorophosphate (LiPF_6_) has been the standard lithium salt for conventional electrolytes, its instability at high voltages necessitates alternatives such as lithium bis(fluorosulfonyl)imide (LiFSI) and lithium bis(trifluoromethanesulfonyl)imide (LiTFSI) [38, 39], which have demonstrated superior stability and compatibility with high-voltage cathodes, offering enhanced ionic conductivity and thermal stability. High-concentration electrolyte formulations, often referred to as “superconcentrated” electrolytes [40–42], have been explored to enhance the stability and performance of high-voltage LMBs by reducing the number of free solvent molecules prone to oxidative decomposition, thus extending the electrolyte stability window and enhancing overall battery performance. The application of electrolyte additives containing fluorine (F) and boron (B) elements in lithium metal batteries has garnered widespread attention [43, 44]. Fluorine is considered a key factor in enhancing the stability of the SEI layer, as it can improve the compactness and conductivity of the SEI, suppress side reactions, and thus significantly enhance the cycling efficiency and overall performance of the battery [45]. At the same time, B, with its unique electronic structure and chemical properties, shows significant potential in improving ionic conductivity, suppressing lithium dendrite growth, and enhancing the mechanical stability of the SEI layer [46]. The synergistic effect of fluorine and boron not only significantly strengthens the stability of the SEI layer but also effectively inhibits the growth of lithium dendrites, thereby extending the battery’s cycle life and enhancing its safety. For example, Zhong utilized a non-flammable ether-based electrolyte and added dual additives of fluoroethylene carbonate and lithium oxalyldifluoroborate, successfully forming a high-modulus SEI rich in F and B species, thereby achieving excellent cycling stability. Additionally, He synthesized an electrolyte additive-tris(2,2,3,3,3-pentafluoropropyl)borane (TPFPB)-for ultra-stable Li||NCM batteries [47]. However, ester-based electrolytes used in Li metal batteries still face challenges under high-voltage conditions. For instance, ethylene carbonate (EC) and diethyl carbonate (DEC) tend to decompose at high voltages, producing unstable by-products. Additionally, Li||Cu cells suffer from low Coulombic efficiency (CE), and the low solubility of LiNO_3_ remains a significant issue. Therefore, optimizing solvent components and proportions, along with incorporating functional additives, is crucial for developing electrolyte formulations suitable for high-voltage Li metal batteries.

**Fig. 1:**
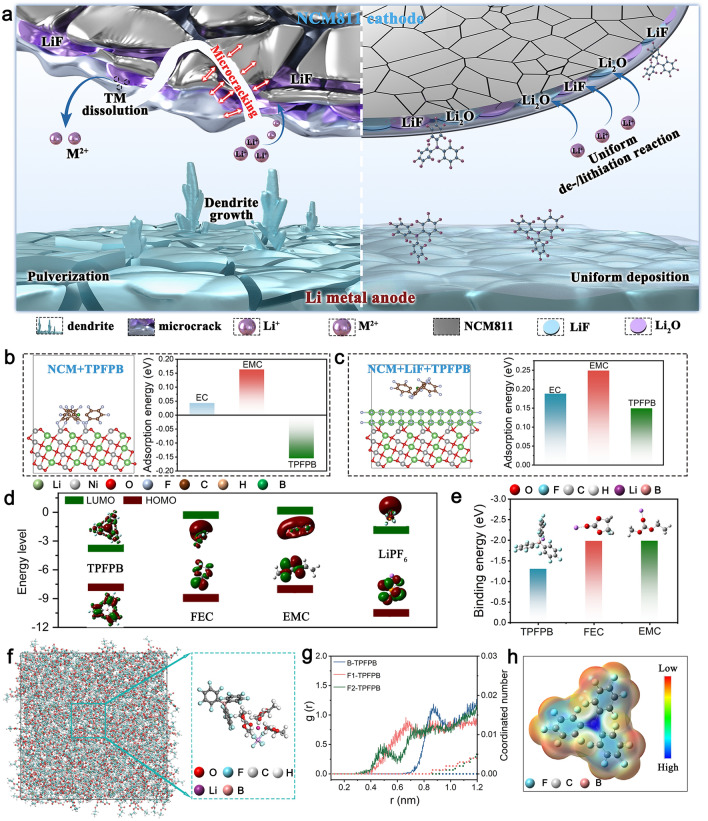
**a** Schematic illustrating the influences of additive-free electrolyte and TPFPB/LiNO_3_ electrolyte on NCM811 cathode and Li metal anode; **b** DFT calculation of H adsorption energy of TPFPB on the surface of NCM811; **c** DFT calculation of adsorption energy of TPFPB on the surface of the NCM811/LiF layer; **d** LUMO and HOMO energy levels of EMC, FEC, TPFPB, and LiPF_6_; **e** Binding energy calculation of EMC, FEC, and TPFPB with Li metal; **f** MD simulation of the electrolyte system with the addition of TPFPB and its enlarged structure; **g** Radial distribution function and coordination number of B and F in different positions of TPFPB molecules; **h** Electrostatic potential density distribution of the TPFPB molecules

This study introduces lithium nitrate (LiNO_3_) and TPFPB additives into an ethyl methyl carbonate/fluoroethylene carbonate (EMC/FEC) electrolyte. This electrolyte design confers several advantages (Fig. 1a): (i) EMC contributes low viscosity and high ionic conductivity, while FEC facilitates stable SEI formation and maintains stability under high-voltage; (ii) the incorporation of LiNO_3_ aids in the formation of a more stable SEI layer, which inhibits lithium dendrite growth and enhances the CE of the batteries; and (iii) TPFPB, a fluorine-rich additive, contains boron and fluorine elements that improve the stability of the SEI and bolster the overall stability of the lithium metal. Furthermore, TPFPB suppresses the oxidative decomposition of the electrolyte at high voltages, thereby forming a protective interfacial layer on the cathode surface, which enhances battery performance under high-voltage conditions. As a result, Li||Cu cells achieve a CE of 98.96%, Li||Li symmetric cells exhibit an impressive lifetime of 4,000 h, and Li||NCM811 full cells retain 87.8% of their capacity after 100 cycles at 4.7 V. Additionally, Li||LNMO full cells demonstrate excellent rate capability, delivering 132.2 mAh g^−1^ at 10 C and retaining 95.0% of their capacity after 250 cycles at 1 C and 5 V. The Li||NCM811 batteries retain 96.1% capacity after 70 cycles at − 20 °C and 87.7% after 100 cycles at 60 °C, showing good low and high-temperature stability. The Li||LNMO battery maintains 89.3% capacity after 100 cycles at − 20 °C and 99.1% at 60 °C, demonstrating strong low-temperature adaptability and excellent high-temperature stability. The Li||NCM811 pouch cells exhibit stable cycling at 4.5 V for 30 cycles, while NCM811||graphite pouch cells retain 93.4% of their capacity after 1100 cycles at 1 C.

## Experimental Section

### Materials

LiPF_6_ (99.9%), LiNO_3_ (99.9%), ethyl methyl carbonate (EMC), fluoroethylene carbonate (FEC), and tris(pentafluorophenyl)borane (TPFPB) were purchased from Aladdin Reagent. LiPF_6_, LiNO_3_ and TPFPB were dried under vacuum at 80 °C for 12 h before use. All solvents and electrolytes were dehydrated using 4 Å molecular sieves for 48 h. 2032-type coin cells, lithium foil (0.45 mm, 99.99%), Al foil (14 μm, 99.6%), Cu foil (16 mm, 99.99%), Cellgard separator (25 μm), and NCM811 cathode and LNMO cathode were obtained from Guangdong Canrd New Energy Technology Co., Ltd. The cathodes were punched into 12-mm disks and dried under vacuum at 80 °C for 12 h before use.

### Preparation of Electrolytes

To prepare electrolyte solutions, a control group and five experimental groups were set up. Each group was first charged with 1500 µL of FEC and 3500 µL of EMC. The components were mixed thoroughly to ensure homogeneity. Subsequently, 0.76 g of 1M LiPF_6_ salt was added to each group, and the mixtures were stirred until the salt was completely dissolved. For the experimental groups, varying amounts of TPFPB were introduced in steps of 0.5% by mass, resulting in final TPFPB concentrations of 0.5%, 1.0%, 1.5%, and 2.0% by mass in the respective groups. Trace amounts of boron trifluoride (BF_3_) were then added to all groups, followed by the addition of lithium nitrate (LiNO_3_) at a concentration of 2.0% by mass. The experiment group with only 2.0% LiNO_3_ added, without TPFPB, was also included. All prepared solutions were stirred at 45 °C for 2 h to ensure complete dissolution and uniform distribution of all components.

### Materials Characterization

To characterize the Li deposited on the Cu substrate, the Li||Cu cells were disassembled in an Ar-filled glovebox and washed with EMC to remove any residual electrolytes. The morphologies of the Li metal anodes and NCM811/LNMO cathodes were examined using a Hitachi SU-8230 field emission scanning electron microscope (SEM). FIB-SEM images were acquired with a Thermo Scientific Helios 5 CX, operating at voltages between 200 V and 30 kV. Transmission electron microscopy (TEM), energy-dispersive X-ray analysis (EDX), and elemental mapping were performed using a Talos instrument at 300 kV. X-ray photoelectron spectroscopy (XPS) was conducted with a Thermo Scientific ESCALAB 250Xi, using Al Kα radiation (*hυ* = 1486.8 eV). Crystalline structures were analyzed via X-ray diffraction (XRD) with a Bruker Advance D8 Ultima IV, using Cu-Kα radiation at a scan rate of 5° min^−1^. Raman spectra were recorded with a HORIBA LabRAM HR Evolution system, using a 532-nm laser. The morphology and Young's modulus of the electrodes before and after cycling were examined via atomic force microscopy (AFM). For Cryo-EM characterization, TEM copper grids with Li deposits were rapidly frozen in liquid nitrogen and transferred to the Cryo-EM (Titan Krios G3i) using a Cryo-transfer box. The Cryo-EM was operated at 300 kV and equipped with a Falcon 3 Direct Electron Detector Camera. The samples were maintained at liquid nitrogen temperature throughout the testing process. All Cryo-EM images were analyzed using Digital Micrograph software, with lattice parameters indexed against databases such as the Materials Project, Crystallography Open Database, and ICDD PDF cards.

### Electrochemical Measurements

All electrochemical tests in this study were conducted using 2032-type coin cells assembled in an argon-filled glovebox, with oxygen and water levels maintained below 0.01 ppm. In the Li||Cu half-cell tests, CEs of Li||Cu cells with various electrolytes were evaluated on a Neware battery testing system. To evaluate the electrochemical stability window, linear sweep voltammetry (LSV) measurements of Li||Al half-cells were conducted at a scan rate of 5 mV s^−1^, over a voltage range of 1 to 6 V, using a CHI760F electrochemical workstation. Electrochemical impedance spectroscopy (EIS) measurements for all Li||Cu cells were carried out after 20 cycles at 0.5 mA cm^−2^ and 0.5 mAh cm^−2^, across a frequency range of 10^5^ to 10^−2^ Hz, using a Solartron 1260 system.

To measure the average CE using Li||Cu cells, 5 mAh of lithium metal was first deposited, followed by a single deposition and stripping cycle. Then, a 20-cycle deposition and stripping process was performed with symmetric cells at 20% DOD (depth of discharge) for 50 mAh. Finally, the lithium was fully stripped. The electrochemical performance was evaluated using a Neware battery test system (Shenzhen, China) at 30 °C. Cyclic voltammetry (CV) curves at different scan rates and EIS measurements from 10^5^ to 10^−2^ Hz with an amplitude of 5.0 mV were obtained using an electrochemical workstation (1470E, Solartron Analytical, UK). The rate performance and cycling performance of the Li||NCM811 and Li||LNMO batteries were tested using the Neware battery testing system.

## Results and Discussion

### Theoretical Calculation and Basic Properties of Electrolytes

Figure 1a visually compares the effects of an additive-free electrolyte and a TPFPB-modified electrolyte on the NCM811 and Li metal. Without additives, the electrolyte interacts with the NCM811 cathode and Li metal anode, potentially causing instability and degradation. This interaction may result in the formation of an unstable SEI layer, dendrite growth on the Li anode, and overall poor cycling stability [48]. The introduction of TPFPB additives facilitates the formation of a stable SEI layer on the Li metal and a cathode/electrolyte interphase on the NCM811 cathode. These more stable interfaces enhance battery performance [49]. Density functional theory (DFT) calculations demonstrate the hydrogen adsorption energies of different electrolyte components on the surface of NCM811. The hydrogen adsorption energies serve as an indicator of the ease with which the electrolyte may decompose on the cathode surface [50–53]. Figure 1b presents the DFT calculation results for the hydrogen adsorption energies of EMC, FEC, and TPFPB on the NCM811 surface. Specifically, the hydrogen adsorption energies are: EC = 0.043 eV, EMC = 0.163 eV, and TPFPB = − 0.156 eV (Fig. S1). Since TPFPB does not contain hydrogen, its adsorption energy on the NCM811 surface indicates that TPFPB is more readily adsorbed by NCM811, leading to its oxidation and the formation of a fluorine/boron-containing CEI layer, which helps stabilize the cathode. Figure 1c presents the adsorption energy calculation results for EMC, FEC, and TPFPB on the NCM811/LiF layer surface, with the adsorption energies being: EMC = 0.185 eV, FEC = 0.249 eV, and TPFPB = 0.149 eV (Fig. S2). After the lithium fluoride (LiF) CEI layer is applied to the cathode surface, EMC and FEC exhibit higher adsorption energies, indicating weaker adsorption on the NCM811/LiF surface (Fig. S2). Conversely, TPFPB demonstrates lower adsorption energy, signifying stronger adsorption, which facilitates the formation of a stable CEI that incorporates F and B elements from TPFPB, thereby further stabilizing the cathode. The LiF-containing CEI on the cathode surface shows that EMC, FEC, and TPFPB are not easily adsorbed onto the LiF-containing CEI, effectively preventing electrolyte oxidation. Figure 1d simulates the reduction and oxidation tendencies of EMC, FEC, TPFPB, and LiPF_6_. The highest occupied molecular orbital (HOMO) energy level of TPFPB (− 7.84 eV) is slightly higher than that of EMC (− 8.08 eV), FEC (− 8.90 eV), and LiPF_6_ (− 10.55 eV). This suggests that TPFPB preferentially oxidizes on the electrode surface, forming a stable interfacial layer that enhances the overall stability and performance of the battery. Additionally, due to its higher HOMO energy level, TPFPB can offer oxidative stability at higher voltages, thereby expanding the operational voltage windows of the battery and improving its energy density and performance. The binding energies of EMC (− 1.979 eV), FEC (− 1.984 eV), and TPFPB (− 1.299 eV) with lithium metal were calculated to assess their interaction strengths (Fig. 1e) from the original state (Fig. S3). EMC and FEC exhibit more negative binding energies with lithium metal, suggesting stronger interactions. In contrast, TPFPB demonstrates less negative binding energy with lithium metal, indicating weaker interactions than EMC and FEC. This weaker interaction of TPFPB with lithium metal facilitates the formation of a stable SEI on the lithium anode by selectively participating in the SEI formation process without excessively reacting with the lithium metal. Molecular dynamics (MD) simulations of the electrolytes and their enlarged structures are presented in Figs. 1f and S4. The MD simulation illustrates the behavior and arrangement of TPFPB molecules in the electrolyte system, showcasing how TPFPB contributes to the overall stability of the electrolyte. The enlarged structure provides a detailed view of the configuration and interactions of TPFPB molecules within the electrolyte, in comparison with the additive-free electrolyte. The radial distribution function (RDF) and coordination number of B and F in different positions of TPFPB molecules are analyzed (Figs. 1g and S5). The RDF reveals the probability distribution of B and F atoms at different distances, highlighting the molecular interactions within the electrolyte. The coordination number indicates the number of neighboring atoms around B and F, providing insights into the structural stability and coordination environment of TPFPB in the electrolyte. TPFPB preferentially occupies the second solvation layer at a distance of 0.4–0.6 nm, without entering the first solvation layer. This is because TPFPB has a complex electronic structure and significant steric hindrance as a large organic molecule, resulting in weaker intermolecular interactions, which makes it more easily excluded from the primary solvent layer and placed in the second solvent layer. TPFPB limits the direct contact between lithium ions and water molecules through a physical steric effect, thereby indirectly inhibiting the formation of lithium dendrites. The electrostatic potential density distribution of TPFPB molecules is illustrated in Fig. 1h. Compared to EMC and FEC (Fig. S6), TPFPB exhibits an electrostatic potential distribution characterized by a central positive region and an outer negative region. This unique distribution enables TPFPB to function effectively as an anion receptor, facilitating interactions with anions. Particularly under high-voltage conditions, these interactions mitigate side reactions at the electrode–electrolyte interface, thereby prolonging battery life. Furthermore, TPFPB plays a role in regulating anion behavior, reducing uneven lithium deposition, and lowering the risk of lithium dendrite formation, thereby enhancing battery safety [54].

**Fig. 2:**
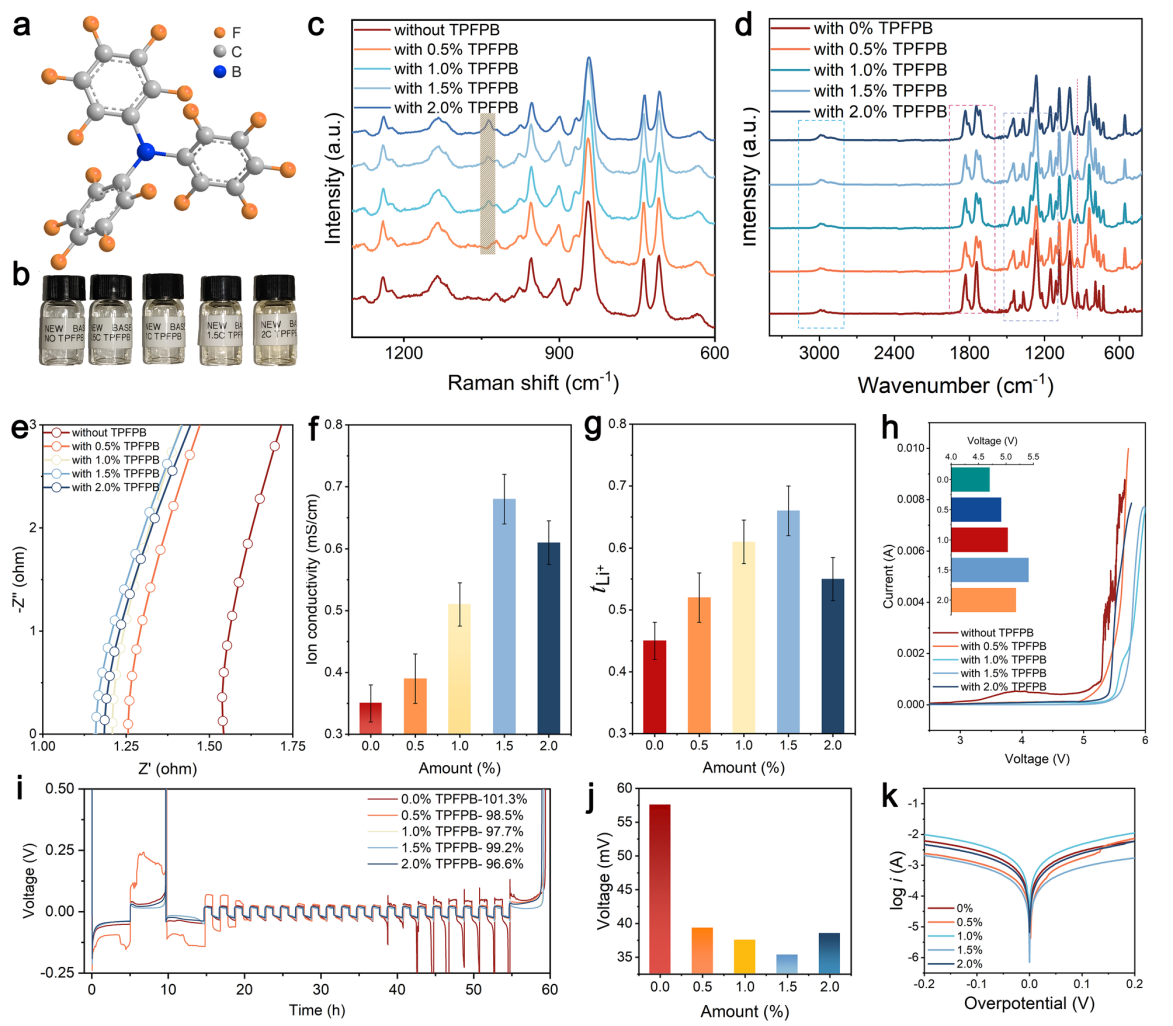
**a** Molecular structure of TPFPB, **b** digital photos of electrolytes with different additive concentrations, **c** Raman spectra of different electrolytes, **d** FTIR spectra of different electrolytes, **e** EIS curves of different electrolytes at 30 °C, **f** ionic conductivity, **g** lithium ion transference number, **h** electrochemical window, **i** average coulombic efficiency, **j** overpotential, **k** Tafel curves of electrolytes with different additive concentrations

To further verify the impact of introducing TPFPB on various properties of the designed electrolyte, we conducted a series of basic physicochemical characterizations and electrochemical tests. Figure 2a illustrates the molecular structure of TPFPB, an additive rich in fluorine and containing boron. Figure 2b presents digital images of electrolytes with varying TPFPB concentrations, demonstrating that the electrolyte gradually adopts a slight yellow hue as the additive concentration increases. Figure 2c, d provides insights into the molecular interactions and chemical composition of different electrolytes through Raman and FTIR spectra. The Raman spectra reveal the characteristic peaks of EMC and FEC solvents. The introduction of TPFPB generates a new peak at 940 cm^−1^, with intensity increasing as the additive concentration rises, indicating alterations in the molecular environment and solvation structure of the electrolyte. This interaction can affect the solubility and ionic conductivity of the electrolytes. Additionally, TPFPB could form a protective barrier, reducing direct contact between solvent molecules and the Li metal electrode, thereby slowing SEI formation and enhancing its stability. The FTIR spectra confirm changes in the vibrations of specific functional groups such as P–F, B–F, and C=O, reflecting interactions between TPFPB and the solvent molecules (Fig. 2d) [55]. These shifts suggest that TPFPB forms strong interactions with the solvent, likely through hydrogen bonding or other polar interactions, which could influence the molecular arrangement and stability of the electrolyte. The EIS curves at 30 °C display the impedance characteristics of the electrolyte, with varying TPFPB concentrations influencing resistance and ion migration. Figure 2e shows that the overall resistance of the electrolyte is minimized at a TPFPB concentration of 1.5%. To characterize the lithium ion conductivity in the electrolyte we designed, Fig. 2f presents the ionic conductivity data, indicating that conductivity reaches 0.68 mS cm^−1^ at 1.5% TPFPB (Table S1). Additionally, the lithium ion transference number is an important indicator that affects the migration capability of lithium ions. To verify the range of potentials over which the electrolyte can remain stable on the electrode surface, Fig. 2g demonstrates that the Li⁺ ion transference number increases with TPFPB concentration, reaching a maximum value of 0.71 at 1.5% TPFPB (Fig. S4, Table S2). To verify the range of potentials over which the electrolyte can remain stable on the electrode surface, Fig. 2h depicts the electrochemical window of the various electrolytes, which expands with increasing TPFPB concentrations, reaching a maximum value of 5.41 V at 1.5%, a critical factor for compatibility with high-voltage cathodes (Table S3). Figure 2i reveals the average CE of the electrolyte during cycling, where higher TPFPB concentrations result in improved efficiency and reduced capacity loss. This indicates that the introduction of TPFPB enhances the stability and performance of the as-designed electrolyte, leading to better efficiency and longevity of high-voltage LMBs. The overpotential measurements indicate that TPFPB reduces energy loss during electrochemical reactions, significantly decreasing polarization during Li metal deposition and stripping processes (Fig. 2j and Table S4). The Tafel curves illustrate the electrochemical kinetics and corrosion behavior, revealing that 1.5% TPFPB enhances kinetics and reduces corrosion rates, thereby contributing to longer battery life and stability (Fig. 2k). These findings demonstrate that introducing TPFPB additives effectively enhances the stability, ionic conductivity, and electrochemical window of the electrolytes for high-voltage LMBs.

### Performance of Li Metal Anode in Designed Electrolytes

**Fig. 3:**
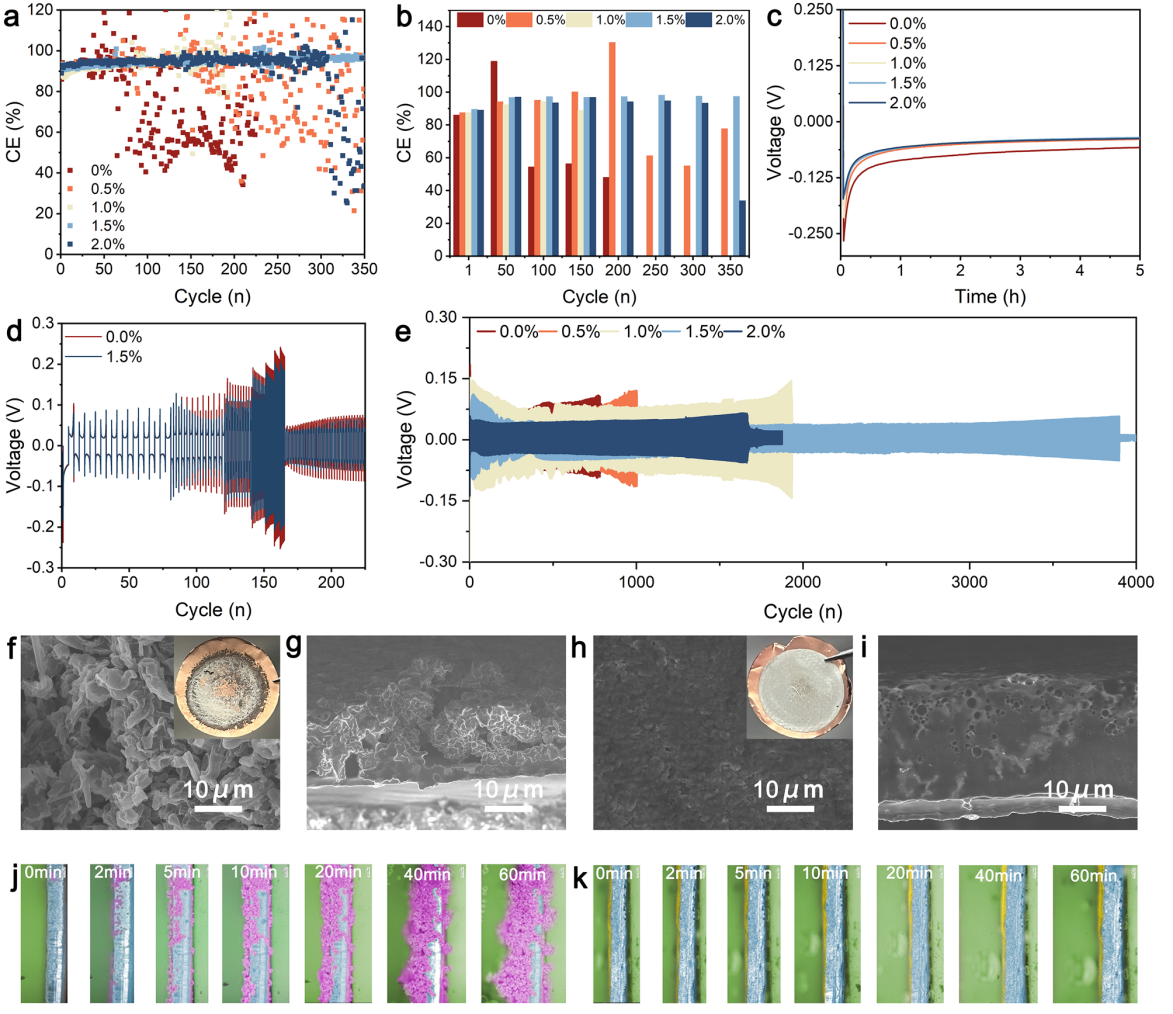
**a** Coulombic efficiency of electrolytes with different additive concentrations in Li||Cu cells, and **b** the corresponding comparison of coulombic efficiency over different cycles for different electrolytes; **c** The first Li deposition curves of different electrolytes; **d** Rate performance of Li symmetric cells with electrolytes without additives and with 1.5% TPFPB additive; **e** Cycling performance of Li symmetric cells with different electrolytes at 30 °C; SEM images and corresponding cross-sections of 1 mAh Li metal deposition **f**, **g** without additives, and **h**, **i** 1.5% TPFPB additive (Inset images are digital pictures); *In situ* optical observation of Li dendrites **j** without additives, and **k** with 1.5% TPFPB additive in the electrolyte

To investigate the interaction between the designed electrolyte and lithium metal, tests were conducted using Li||Cu half-cells and Li||Li symmetric cells. Figure 3a presents the CE of electrolytes with varying concentrations of TPFPB in Li||Cu cells. As the concentration of TPFPB increases, the CE values gradually improve and stabilize, underscoring the crucial role of TPFPB in enhancing the stability of the interface between the electrolyte and Li metal electrodes. The formation of a stable SEI layer effectively prevents further electrolyte decomposition, reduces electrochemical reactions on the electrode surface, and mitigates battery polarization [56]. By comparing the CE of various electrolytes over multiple cycling periods, the initial CE reaches 89.0%, stabilizes at 98.5% after 50 cycles with 1.5% TPFPB, and maintains 98.9% even after 350 cycles. In contrast, we first compared the electrochemical performance of Li metal anodes in electrolytes with and without LiNO_3_, as shown in Fig. S8. The basic electrolyte without LiNO_3_ additive shows a lower CE of around 93.0% at 0.5 mA cm^−2^ and 0.5 mAh cm^−2^. The electrolyte with 2.0% LiNO_3_ additive exhibits a significant improvement in CE, reaching around 97%. This suggests that LiNO_3_ as an additive effectively enhances the electrochemical stability of the electrolyte, by promoting the formation of a stable solid electrolyte interphase layer. This process helps reduce the polarization of the lithium metal electrode, minimize side reactions, and improve cycling efficiency. The baseline electrolyte without TPFPB shows significant polarization after 50 cycles, highlighting the critical role of TPFPB in enhancing and maintaining SEI layer stability (Fig. 3b). Figure 3c presents the initial Li deposition curves of various electrolytes, reflecting the nucleation and stable potentials of Li metal deposition under different electrolyte conditions. Notably, the electrolyte containing 1.5% TPFPB exhibits a lower overpotential of 37.5 mV compared to other concentrations, indicating superior kinetic efficiency during lithium ion reduction and deposition processes in the presence of 1.5% TPFPB. Figure 3d provides a detailed study of the performance of Li||Li symmetric cells under different plating/stripping rates using 1.5% TPFPB electrolyte and an additive-free electrolyte. In contrast, the cycling stability of Li|Li batteries at 0.25 mA cm^−^^2^ and 0.25 mAh cm^−^^2^ shows that without the LiNO_3_ additive, the polarization is relatively high, around 20.0 mV. However, with the addition of 2.0% LiNO_3_, the polarization decreases to approximately 14 mV (Fig. S9). These results demonstrate that TPFPB significantly enhances the rate capability of the cells. A comparison of polarization voltage at different rates shows lower overpotential in the TPFPB-added electrolyte, particularly at 10 mA cm^−2^ where the polarization voltage is reduced to 120 mV from 160 mV observed in the additive-free electrolyte (Fig. S10). This underscores the role of TPFPB in improving the Li⁺ ion transference number and ion conductivity, thereby enhancing the rate of Li⁺ ion conduction.

Figure 3e depicts the cycling performance of various electrolytes in Li symmetric cells at 30 °C. The data reveal that the electrolyte with 1.5% TPFPB exhibits remarkable stability over extended cycles, maintaining a low polarization voltage of 66 mV over 3,900 h at 1.0 mA cm^−2^ and 1.0 mAh cm^−2^. In contrast, the baseline electrolyte without additives shows a significant increase in polarization voltage after 200 h, reaching 300 mV after 780 h and eventually leading to short-circuiting, indicating poor cycling stability. Increasing the TPFPB concentration progressively reduces the deposition and stripping polarization voltages, thereby extending cycle life, with optimal performance observed at a 1.5% additive concentration. As the depositing and stripping processes continue, the Li||Li cells still maintain low polarization and stable cycling, as shown in the enlarged curves (Fig. S11). This highlights the significant role of TPFPB in enhancing the interface stability between the electrolyte and Li metal electrodes, suppressing Li dendrite formation, and greatly improving battery cycling performance.

Figure 3f–i presents SEM images of deposited Li metal. The illustrations in Fig. S12 depict optical images of lithium metal deposited on the surface of copper foil. Compared to the uneven deposition observed without additives, the introduction of TPFPB leads to a uniform, dense, and smooth lithium morphology on the copper (Cu) surface (Fig. S13). Figure 3f, g displays lithium metal deposited in electrolytes without additives, exhibiting pronounced dendritic structures indicative of uncontrolled growth. Cross-sectional SEM images further reveal a loose and multi-dendritic deposition morphology. In contrast, Fig. 3h, i illustrates lithium metal deposition in electrolytes containing 1.5% TPFPB, demonstrating more uniform deposition with significantly reduced dendritic formation, thereby underscoring TPFPB's role in effectively suppressing dendrite growth. Finally, in situ optical observations of lithium dendrite formation and evolution in electrolytes with and without the 1.5% TPFPB additive are presented in Fig. 3j, k, respectively. In the absence of the additive (Fig. 3j), typical dendritic structures are observed, growing unevenly on the electrode surface over time and potentially leading to short circuits. Conversely, in the presence of 1.5% TPFPB (Fig. 3k), deposited lithium appears more uniform with markedly reduced dendrite formation, demonstrating TPFPB's significant role in inhibiting dendrite growth and enhancing interface stability between the electrolyte and lithium metal electrodes.

### SEI Interface Characterizations in Designed Electrolytes

**Fig. 4:**
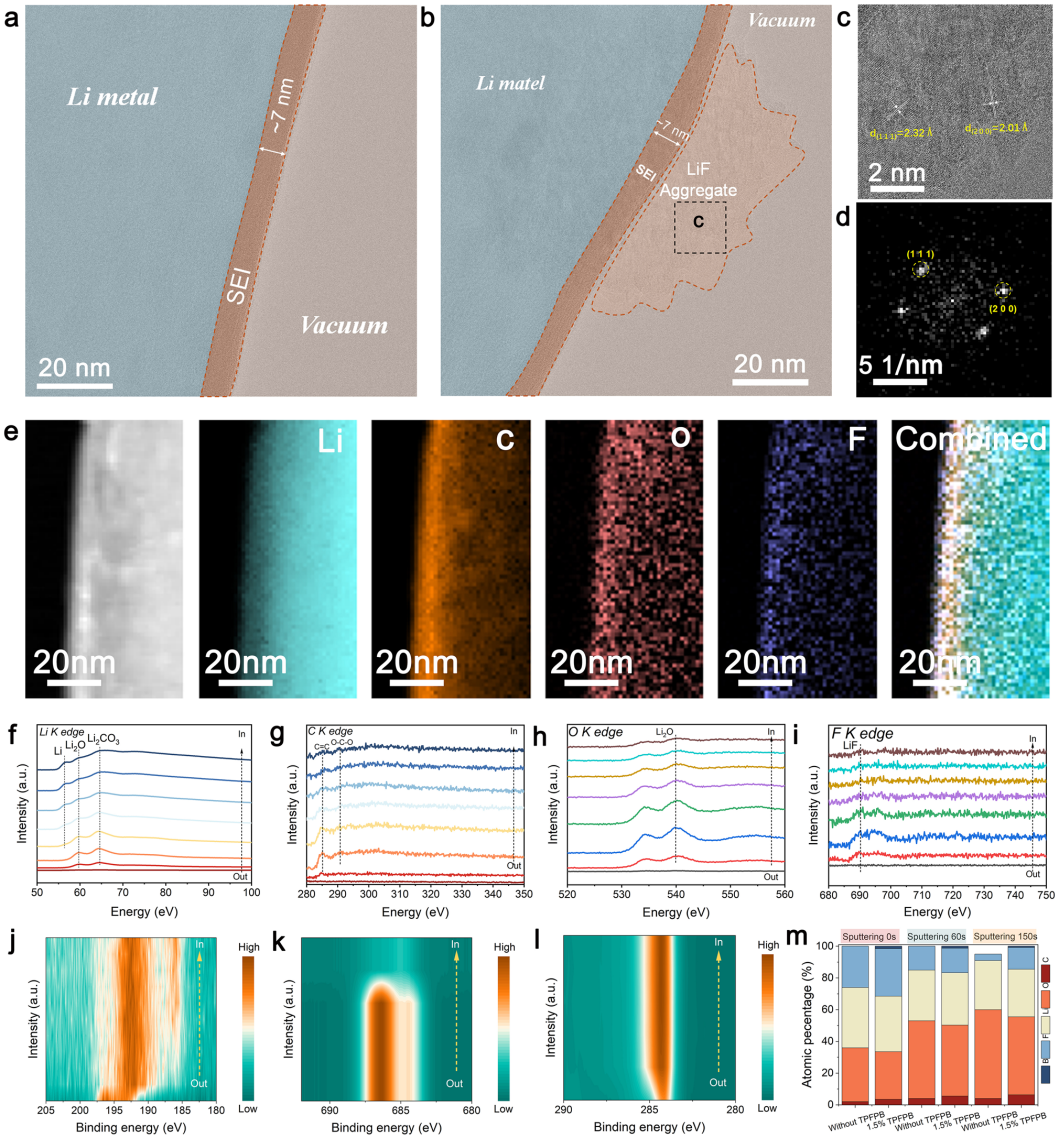
**a** Cryo-TEM images of the SEI formed at 30 °C. **b** High-resolution Cryo-TEM images of the SEI with TPFPB additive. **c** Enlarged topographies of local areas in (**b**). **d** FFT pattern of the selected area in (**b**). **e** EELS mapping and **f**–**i** EELS line scan of element distribution in the SEI. **j**–**l** XPS spectra of B 1*s*, F 1*s*, and C 1*s* for the SEI formed on deposited Li after 5 cycles in Li||Cu asymmetrical cells with modified electrolytes. **m** Atomic ratios of Li 1*s*, C 1*s*, B 1*s*, O 1*s*, and F 1*s* in the SEI at different sputtering times

To investigate the impact of the TPFPB additive in electrolytes on the formation and properties of the SEI layer, we deposited lithium metal on a copper mesh by assembling Li||Cu cells with the additive-free electrolyte and TPFPB-modified electrolyte. Using cryogenic transmission electron microscopy (cryo-TEM), we obtained high-resolution images to analyze the sensitive materials, revealing detailed information about the morphology, lattice ordering, and nanostructures of the SEI, along with the interface structures between the SEI and the deposited lithium (Fig. 4). In the additive-free electrolyte, the SEI displays a non-uniform morphology with a thickness of about 60 nm. The outer layer predominantly comprises Li_2_CO_3_, Li_2_O, and LiF, while it is enveloped by an amorphous phase (Fig. S14). This outer SEI layer continuously reacts with the electrolyte, producing CO_2_ gas and evolving into a spongy framework [57]. In contrast, the SEI layer formed in the TPFPB-modified electrolyte exhibits a smooth morphology with a thickness of around 7 nm (Fig. 4a), featuring an amorphous phase and uniformly distributed LiF nanoclusters (Figs. S15 and 4b). High-resolution cryo-TEM and fast Fourier transform (FFT) analysis confirmed the crystal plane spacings of the LiF (111) crystal (2.32 Å) and the (200) crystal (2.01 Å) (Fig. 4c), consistent with bulk LiF in the crystal structure database (Fig. 4d).

To further analyze the composition of the inorganic and amorphous phases of the SEI, we utilized electron energy loss spectroscopy (EELS) for elemental mapping. As depicted in Fig. 4e, the SEI layer with the TPFPB-modified electrolyte contains abundant F-, O-, and C-based compounds, originating from the LiTFSI and LiNO_3_. Li, C, O, and F elements are uniformly distributed in the SEI formed with the TPFPB additive, indicating effective and even coverage of the lithium metal, which effectively minimizes additional interactions between lithium and electrolytes. The gradient distribution of F and O elements from the outer to the inner part of the SEI is confirmed by EELS K-edge results (Fig. 4f-i). Specifically, the content of Li_2_O and LiF gradually decreases, indicating an SEI composition rich in inorganic materials. Additional morphological examinations through SEM and AFM highlight notable contrasts in the deposited lithium using 0.5 mA cm^−2^ and 2.0 mAh cm^−2^. In the additive-free electrolyte, the lithium deposits show a textured and porous surface (Fig. S16a), whereas in the TPFPB-modified electrolyte, it displays a smooth columnar structure (Fig. S16b). The 3D-AFM analysis of the lithium deposited from the additive-free electrolyte shows a porous and irregular morphology with complex dendrites and a height variation of roughly 3.5 μm (Fig. S17a). Conversely, lithium deposited from the TPFPB-modified electrolyte shows a more uniform structure with micron-sized grains with height variations of less than 0.23 μm (Fig. S17b). These results highlight the critical impact of a well-structured SEI on ensuring even lithium deposition, which greatly enhances both the stability and efficiency of the lithium metal anodes.

XPS was utilized to further investigate the chemical composition of the SEI formed in the two different electrolytes. As shown in Figs. 4j–l and S18, the SEI formed in both electrolytes exhibited similar components, including Li_2_O, LiF, Li_2_CO_3_, and C-containing organic compounds. Following Ar-ion sputtering treatment, a reduction in the content of organic components was observed in both types of SEI (Fig. S18), indicating that these components are predominantly distributed in the outer region of the SEI. Additionally, comparative analysis of the F 1*s* spectra showed that LiF formed in the presence of TPFPB is uniformly distributed throughout the entire SEI (Fig. 4k). Furthermore, the N 1*s* spectra demonstrated the presence of nitrogen-containing compounds such as LiN_x_O_y_ and Li_3_N within the SEI layer, originating from the decomposition of FSI^−^ anions (Fig. S19). Only minor differences were observed in the composition of the SEI layers for the two electrolytes, likely due to their similar electrolyte composition. Therefore, the distinct electrochemical performances and lithium deposition morphologies observed in these two electrolytes can be attributed to differences in SEI structure, which are significantly influenced by interfacial reaction processes. A deep understanding of the interfacial reaction kinetics during SEI formation is thus essential for exploring the properties of the SEI formed in these two electrolytes. Analysis of the elemental ratios at different etching depths revealed a higher F content and surface enrichment of B in the modified electrolyte (Fig. 4m), which is crucial for stabilizing the SEI structure and enhancing battery stability.

### Electrochemical Performance of Li||NCM811 and Li||LNMO Full-Cells

**Fig. 5:**
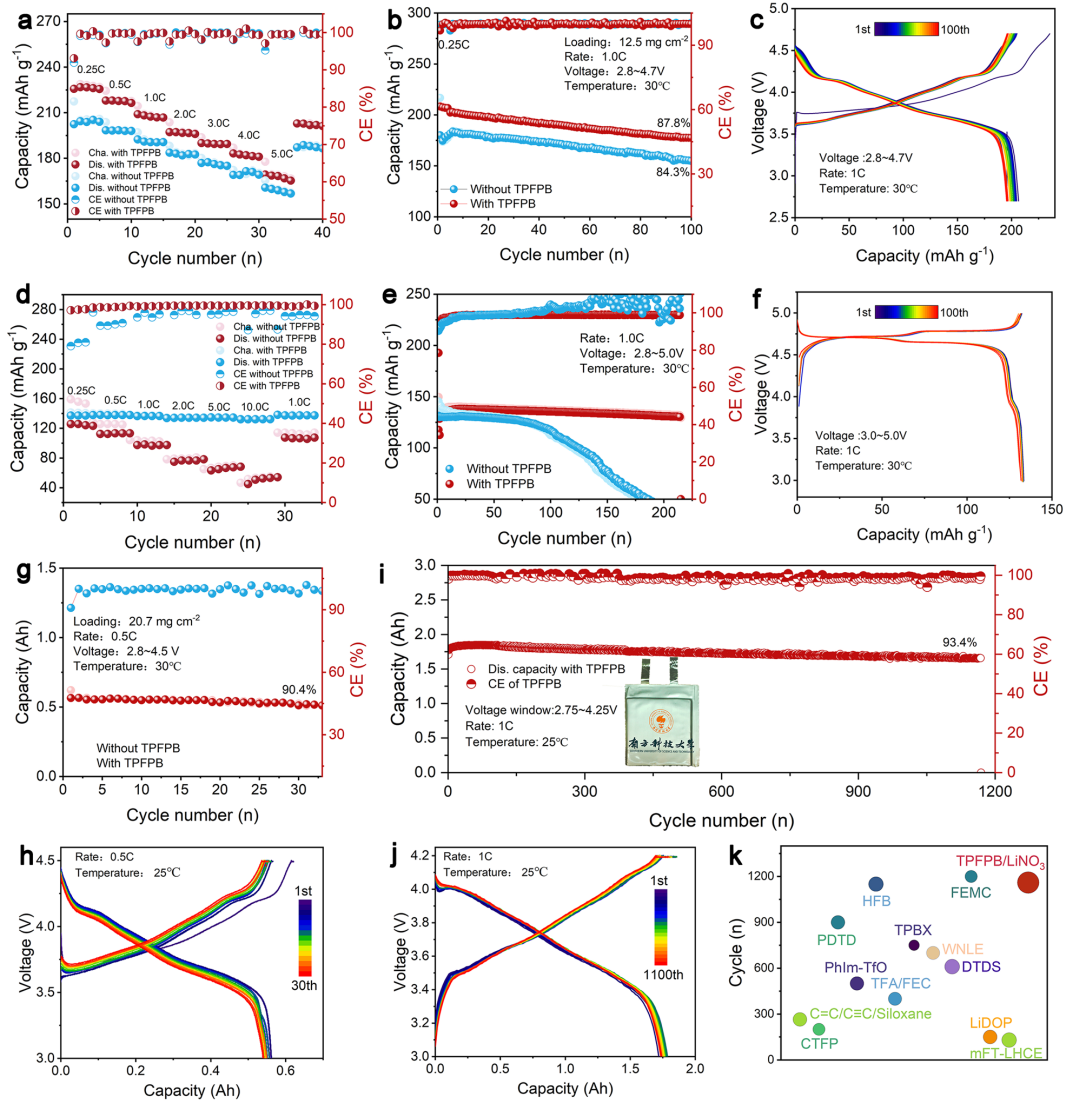
**a** Rate performance, **b** cycling performance at 1 C with additive-free and 1.5% TPFPB additive electrolytes; **c** Charge–discharge curves for the first 100 cycles of NCM811 at 4.7 V with 1.5% TPFPB additive electrolyte; **d** Rate performance, **e** cycling stability at 1 C with 1.5% TPFPB additive electrolyte; **f** Charge–discharge curves for the first 100 cycles of LNMO at 5 V with 1.5% TPFPB additive electrolyte; **g** Cycling stability of Li||NCM811 pouch cell at 0.5 C; **h** the corresponding charge–discharge curves at 4.5 V high voltage; **i** Cycling stability of graphite||NCM811 pouch cell at 1 C; **j** The corresponding charge–discharge curves; **k** Performance comparison of graphite||NCM811 pouch cell with reported functional electrolytes (the size of the circle indicates the capacity retention)

To investigate the effects of the TPFPB-modified electrolyte in high-voltage LMBs, the Li||NCM811 and Li||LNMO cells were assembled to examine the electrochemical performance and mechanism of TPFPB under high voltages. As shown in Fig. 5a, the rate performance of Li||NCM811 cells demonstrates that cells with the TPFPB-modified electrolyte achieve a higher charge and discharge capacity of 226.8 mAh g^−1^ under 4.7 V, maintaining better capacity retention (168.3 mAh g^−1^ at 5 C) across a range of current densities compared to those with additive-free electrolytes (157.8 mAh g^−1^ at 5 C) (Table S5). The corresponding charge–discharge curves further reveal that the TPFPB-modified batteries exhibit less voltage drop and more stable capacity at elevated rates (Fig. S20), indicating enhanced ionic conductivity and reduced internal resistance (Fig. S21). The cycling performance at 1 C shows a marked improvement in stability with the addition of 1.5% TPFPB under a high cathode loading of 14.0 mg cm^−2^, as the battery exhibits significantly less capacity fade over 100 cycles, with a capacity retention of 87.8% (Fig. 5b). Charge–discharge curves for the first 100 cycles of NCM811 at 4.7 V with 1.5% TPFPB additive electrolyte reveal consistent and overlapping curves, indicating stable voltage profiles and minimal capacity loss, thereby reflecting enhanced electrochemical stability (Fig. 5c). Furthermore, LiNO_3_ plays a crucial role. It stabilizes the electrolyte, mitigates side reactions at high voltages, and thus improves the cycling stability of the NCM811 cathode. In contrast, the Li||NCM811 cells with the additive-free electrolyte exhibit greater capacity fade and lower discharge specific capacity. To verify the wide temperature range performance of the electrolyte, we tested the cycling stability of Li|NCM811 and Li|LNMO batteries at both high (60 °C) and low (− 20 °C) temperatures. As shown in Fig. S22, the Li|NCM811 battery maintains a capacity retention of 96.1% after 70 cycles at − 20 °C, demonstrating good low-temperature stability. At 60 °C, after 100 cycles, the capacity retention is 87.7%, showing good high-temperature performance. To further validate the versatility of the designed high-voltage electrolyte, its performance was also investigated in Li||LNMO cells. The rate performance of the Li||LNMO cell demonstrates improved rate capability with the TPFPB additive, enabling the cells to maintain higher capacities at increased charge and discharge rates of 5 and 10 C, respectively (Fig. 5d and Table S6). The Li||LNMO cell with TPFPB-modified electrolyte delivers a capacity of 137.2 mAh g^−1^ at 0.1 C, 134.4 mAh g^−1^ at 5 C, and 132.2 mAh g^−1^ at 10 C. In contrast, the Li||LNMO cell with additive-free electrolyte achieves only 53.3 mAh g^−1^ at 10 C. This significant difference indicates that the TPFPB additive enhances the high-rate performance of the electrolyte, likely by improving ionic conductivity and stabilizing the electrode–electrolyte interface. The cycling stability of the Li||LNMO cell at 1 C with 1.5% TPFPB additive electrolyte demonstrates excellent long-term durability and reliability, retaining 95.3% of its capacity over 210 cycles (Fig. 5e). The Li|LNMO battery retains 89.3% capacity after 100 cycles at − 20 °C, indicating strong low-temperature adaptability (Fig. S23a). At 60 °C, the capacity retention is as high as 99.1%, showing almost no degradation and excellent high-temperature stability (Fig. S23b). In contrast, the Li||LNMO cell with additive-free electrolyte begins to show capacity degradation after 70 cycles, with the capacity dropping to nearly zero around 170 cycles. Charge–discharge curves for LNMO at 5 V with 5% TPFPB additive electrolyte show stable profiles, indicating effective high-voltage management and mitigation of electrolyte decomposition (Fig. 5f). The cycling stability of the Li||NCM811 pouch cell at 0.5 C demonstrates prolonged cycle life, with the cell retaining a high capacity over 30 cycles (Fig. 5g). Corresponding charge–discharge curves at a 4.5 V high voltage indicate stable voltage profiles and consistent performance, further confirming the efficacy of the TPFPB additive in high-voltage operations (Fig. 5h). The cycling performance of the graphite||NCM811 pouch cell at 1 C (Fig. 5i) and the corresponding polarization curves (Fig. 5j) show stable voltage profiles and an extended cycle life of 1160 cycles with a capacity retention of 93.4%, reinforcing the enhanced electrode stability provided by TPFPB. In contrast, the retention of the graphite||NCM811 pouch cell without additives is 81.6% after 455 cycles at 1 C, indicating more rapid capacity degradation (Fig. S24). This finding is further confirmed by the charge and discharge curves, which clearly show a decline in capacity with increasing cycles (Fig. S25). Therefore, a performance comparison of the graphite||NCM811 pouch cell with reported functional electrolytes indicates that the TPFPB additive results in superior capacity retention, demonstrating its effectiveness in enhancing overall battery performance and longevity (Fig. 5k and Table S7).

### Structural Evolution Mechanism and Interface Properties of Cathodes

**Fig. 6:**
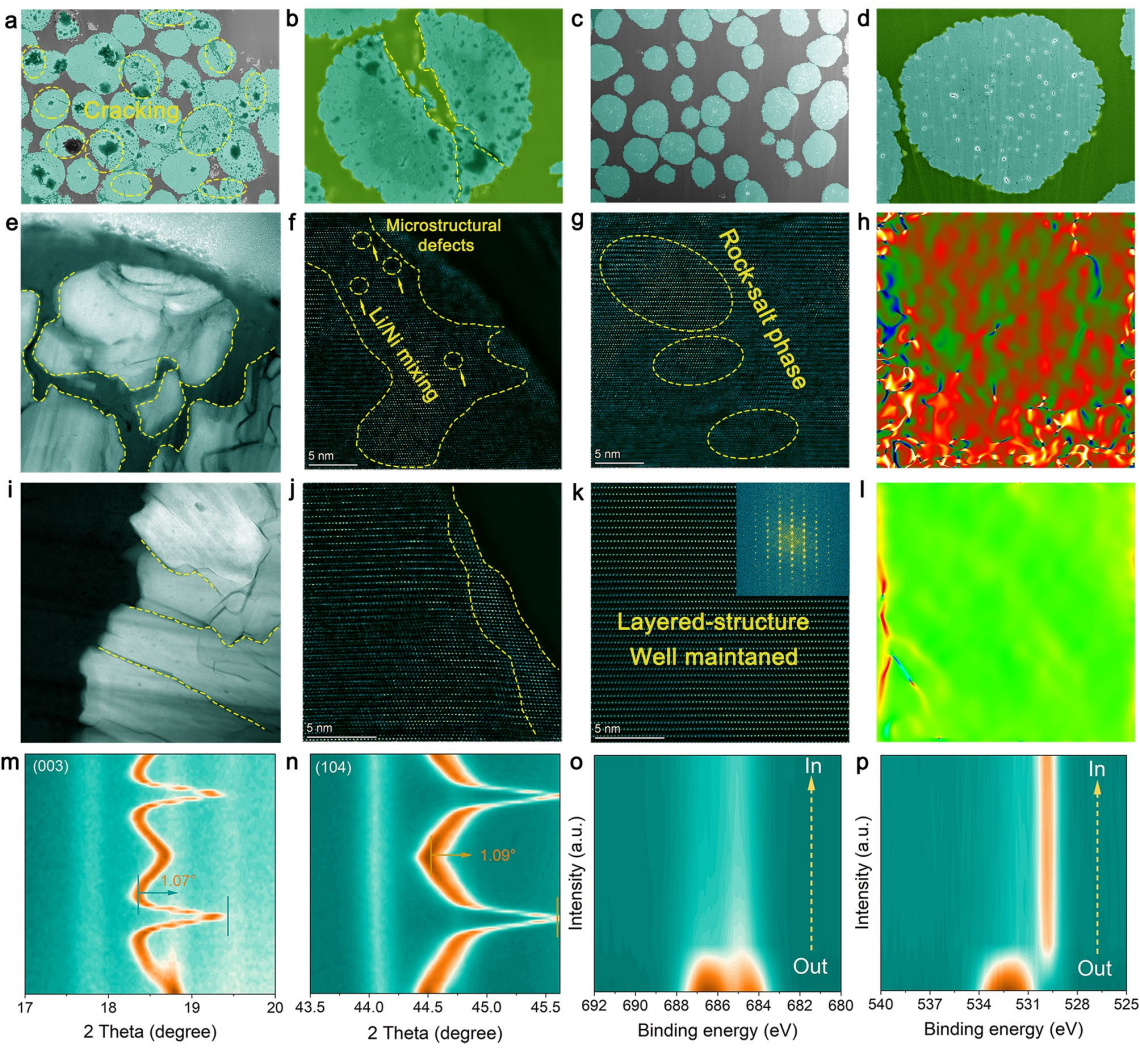
SEM images of NCM811 particles after 100 cycles in the voltage range of 2.7–4.7 V: **a**, **b** with additive-free electrolyte and **c**, **d** with TPFPB-modified electrolyte. TEM images of NCM811 particles after cycling: **e** Low-magnification with additive-free electrolyte, **f** HRTEM of surface region, **g** HRTEM of central region, **h** the corresponding *E*_*xx*_ strain map obtained by performing GPA pattern of **g**, **i** low-magnification with TPFPB additive, **j** HRTEM of surface region, **k** HRTEM of central region, **l** the corresponding *E*_*xx*_ strain map obtained by performing GPA pattern of **k**. **m**, **n** In situ XRD patterns of NCM811 during the initial cycle at 0.1 C between 2.7 V and 4.7 V for (003) and (104) reflections. XPS depth profile spectra of cycled NCM811 particles, **o** C 1*s*, **p** F 1*s*

Building on the enhanced performance of high-voltage LMBs, we conducted detailed characterization and analysis of the NCM811 particles cycled for 100 cycles at 4.7 V to investigate the structural evolution of the positive electrode material and the cathode/electrolyte interface. SEM images of NCM811 particles using additive-free electrolyte after 100 cycles at 4.7 V reveal significant surface cracking and particle aggregation (Fig. S26). This surface degradation indicates severe structural instability under cycling conditions without additives. Figure 6a, b present cross-sectional SEM images of cycled NCM811 electrodes prepared using cryo-precision ion milling technology. Substantial cracks and noticeable particle breakage are highly prominent, resulting in the loss of electrical connectivity between particles and an increased interfacial area with the electrolyte, which leads to electrolyte decomposition and gas generation factors contributing to performance degradation and cell swelling [58]. In contrast, SEM images of NCM811 particles cycled with TPFPB-modified electrolyte display a more uniform surface with fewer signs of degradation and reduced particle aggregation (Fig. S27).

The cross-sectional SEM images reveal the structural integrity of NCM811 particles from the exterior to the interior (Fig. 6c, d), indicating that the TPFPB additive effectively constructs stable NCM811/electrolyte interfaces, mitigates particle degradation, and maintains structural integrity under high-voltage conditions. TEM images further detail the internal structure of the NCM811 particles. Figure 6e presents a low-magnification TEM image of NCM811 particles cycled with additive-free electrolyte, revealing severe structural damage and cracking. In contrast, Fig. 6i presents a low-magnification TEM image of NCM811 particles cycled with TPFPB-added electrolyte, where the internal structure is more intact with fewer disordered regions, reflecting better preservation of particle morphology and internal structure. High-resolution TEM (HRTEM) images provide additional insights into the crystal structure of the NCM811 anode. Figure 6f, g present HRTEM images of the surface and central regions of NCM811 particles cycled with additive-free electrolyte, revealing significant lattice degradation and the formation of amorphous regions, consistent with the SEM results. This suggests that the failure of the cathode/electrolyte interface causes structural degradation of the cathode particles, with substantial rock-salt and mixed-phase regions and numerous micro-defects, likely due to transition metal dissolution and structural degradation during cycling. In contrast, Fig. 6j, k presents HRTEM images of the surface and central regions of NCM811 particles cycled with TPFPB-modified electrolyte, where the lattice structure is well-preserved, with a thinner rock-salt phase on the surface (~ 4 nm) and a well-maintained layered structure in the central region, indicating the effectiveness of TPFPB in maintaining the structural integrity of the cathode particles. The geometric phase analysis (GPA) images in Fig. 6h, l further corroborate the stability observed in HRTEM. The GPA image of the additive-free electrolyte reveals high internal strain, indicating significant structural and chemical changes during cycling (Fig. 6h). In contrast, the GPA image of the TPFPB-modified electrolyte exhibits significantly reduced internal strain, consistent with improved particle stability (Fig. 6l). This suggests that the TPFPB additive effectively alleviates strain induced by cycling, thereby enhancing the structural stability of the NCM811 cathode.

In situ XRD patterns in Fig. 6m, n display the (003) and (104) reflections of the NCM811 cathode during the initial cycle. The corresponding charge–discharge curve is shown in Fig. S28. The XRD patterns reveal that NCM811 cycled with TPFPB-modified electrolyte retains good crystallinity and undergoes minimal structural changes. Specifically, the (003) and (104) diffraction peaks of TPFPB-modified NCM811 shift to the right by 1.07° and 1.09°, respectively. This suggests that the TPFPB additive effectively slows the phase transitions at high cutoff voltage (4.7 V) and enhances the reversibility of the H2-H3 phase transition [59]. The XPS depth profile spectra in Fig. 6o, p show the surface composition of NCM811 particles after cycling. NCM811 particles cycled with TPFPB-modified electrolyte exhibit more stable C 1*s* and F 1*s* signals, with a significant increase in the LiF signal at greater depths, combined with abundant amorphous carbon. This indicates the formation of a stable inorganic/organic composite CEI layer on the surface of NCM811 particles, which effectively suppresses electrolyte decomposition and surface degradation of the cathode particles [60]. This enhancement effectively mitigates degradation and maintains particle integrity under high-voltage conditions, thereby improving battery performance and longevity.

**Fig. 7:**
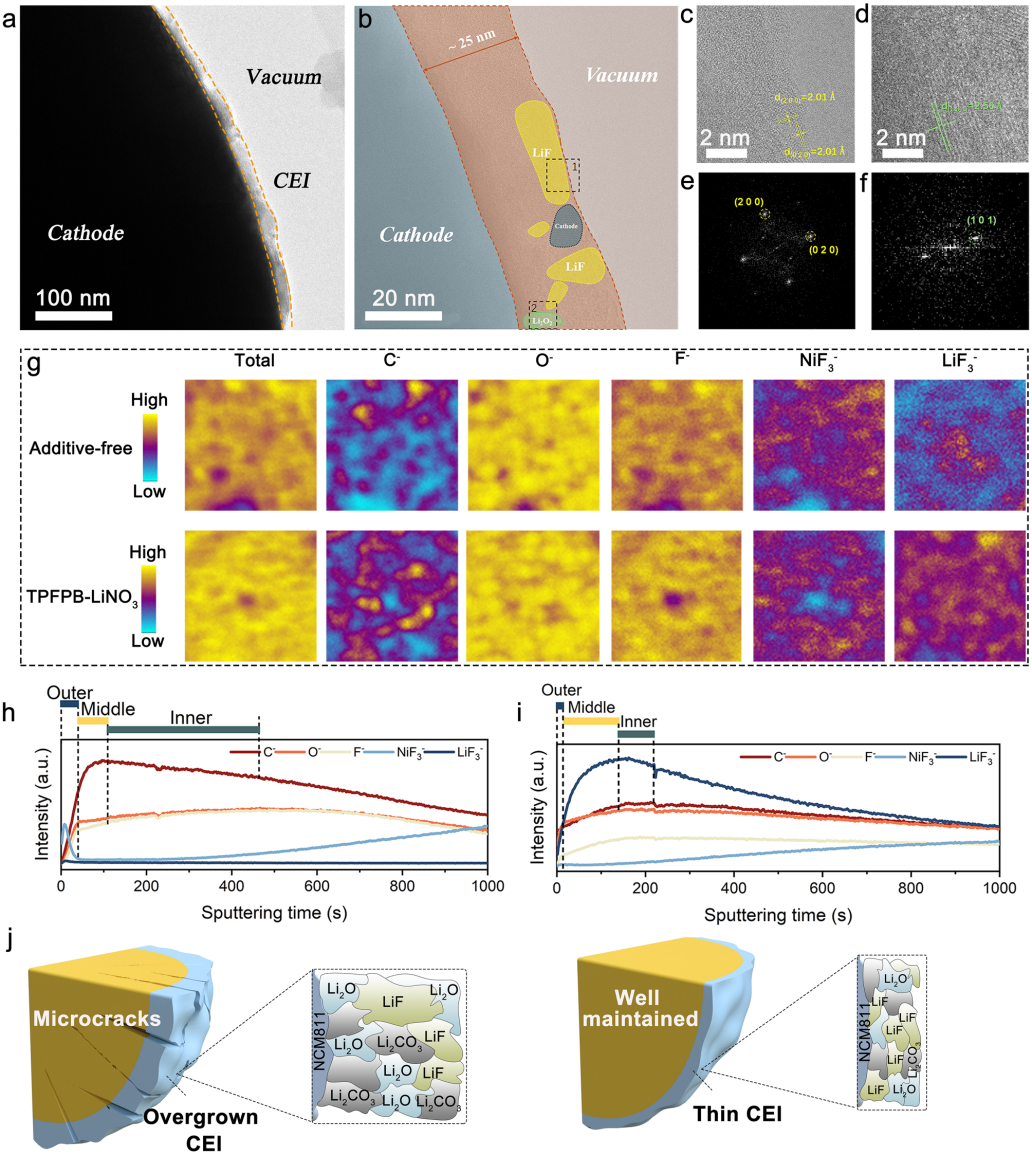
Cryo-TEM images of the CEI film formed at 30 °C: **a** Low magnification cryo-TEM images; **b** High-resolution cryo-TEM image of the CEI formed in TPFPB additive electrolyte. **c**, **d** Enlarged topographies of local areas in (**b**). **e**, **f** Corresponding fast Fourier transfer (FFT) patterns of the selected areas in (**c**) and (**d**). **g** TOF–SIMS 2D images for C^−^, O^−^, F^−^, NiF_3_^−^, and LiF_3_^−^ with additive-free electrolyte and TPFPB/LiNO_3_-modified electrolyte. **h**, **i** Corresponding depth curves. **j** Schematic diagrams of the CEI growth in both electrolytes

To obtain detailed information about the CEI film formed on the NCM811 cathode at 30 °C, we employed cryo-TEM and time-of-flight secondary ion mass spectrometry (TOF–SIMS) imaging techniques to illustrate the structural and compositional differences influenced by electrolyte additives. The low-magnification cryo-TEM image provides an overview of the CEI film structure formed on the NCM811 electrode surface, revealing a uniform and continuous distribution of the CEI layer (Fig. 7a). The high-resolution cryo-TEM image shows that the CEI formed with TPFPB additive is consistent and seamless, with a thickness of 25 nm (Fig. 7b), whereas the CEI formed with additive-free electrolyte consists of disordered, agglomerated particles with a thickness of approximately 55 nm (Fig. S29). A high-resolution TEM image of LiF is shown in Fig. 7c, corresponding to the (200) (2.01 Å) and (002) (2.01 Å) crystal planes, while Fig. 7e displays the corresponding fast Fourier transform (FFT) pattern of LiF. Figure 7d presents an HRTEM image of lithium peroxide (Li_2_O_2_) and Li_2_O, corresponding to the (101) (2.56 Å) crystal plane, and Fig. 7f shows the corresponding FFT pattern of Li_2_O_2_/Li_2_O. These results indicate that the CEI layer formed with TPFPB-modified electrolyte consists of amorphous regions encapsulating LiF and Li_2_O_2_/Li_2_O crystallites, forming a consistent and seamless protective layer. This structure not only enhances the mechanical strength and chemical stability of the CEI but also effectively prevents further decomposition of the electrolyte and the occurrence of side reactions, thereby significantly improving the cycling performance and lifespan of the battery.

Two-dimensional TOF–SIMS images further corroborate the distribution of anionic groups in the CEI layers formed with additive-free and TPFPB-modified electrolytes. Figure 7g shows the distribution of C^−^, O^−^, F^−^, LiF_3_^−^, and NiF_3_^−^ species within the CEI layer. The results indicate that both electrolytes enrich the surface with O^−^ and F^−^ species, but the CEI formed with TPFPB-modified electrolyte shows a significantly higher content of C^−^, with LiF_3_^−^ and NiF_3_^−^ more uniformly distributed on the surface, whereas the CEI with additive-free electrolyte contains relatively less. This suggests that the TPFPB-modified electrolyte forms a CEI layer enriched with more O^−^ and F^−^ species, further demonstrating its superior performance in inhibiting electrolyte decomposition and protecting the electrode. Figure 7h, i show depth profiling curves, indicating the trend of anionic intensity variation. The images reveal that the CEI layer formed without additives is thicker, while the CEI layer formed with TPFPB-modified electrolyte displays distinct external and central regions, with F^−^ and LiF_3_^−^ showing a clear gradient distribution. This suggests that the TPFPB-modified electrolyte not only forms a thinner, more uniform CEI layer, but also that the chemical composition distribution at different depths is more rational, contributing to the overall enhancement of battery performance and stability. The growth mechanisms of the CEI in both electrolyte systems are illustrated in Fig. 7j. In the additive-free electrolyte, the CEI tends to form a more irregular, thick, and less protective layer, prone to decomposition and structural instability. In contrast, the TPFPB/LiNO_3_ additives promote the development of a more robust, uniform, thinner, and protective CEI, enhancing the electrochemical stability and performance of the NCM811 cathode.

## Summary

In conclusion, we have demonstrated that the incorporation of TPFPB as an electron acceptor into an EMC/FEC electrolyte significantly enhanced the performance of high-voltage LMBs. This electrolyte contributed to the formation of durable dual interfaces on both the Li metal anode and the NCM811 cathode, effectively mitigating dendrite formation and improving stability. As a result, the Li metal batteries operated at voltages up to 4.7 V, with Li||Cu cells achieving a CE of 98.96% and Li||Li symmetric cells extending their cycle life to 4,000 h. Additionally, Li||NCM811 full cells maintained 87.8% of their capacity after 100 cycles at 4.7 V, while Li||LNMO full cells delivered 132.3 mAh g^−1^ at 10 C and retained 95.3% capacity after 250 cycles at 1 C with a high-voltage of 5 V. The Li||NCM811 batteries retain 96.1% capacity after 70 cycles at − 20 °C and 87.7% after 100 cycles at 60 °C, showing good low and high-temperature stability. The Li||LNMO battery maintains 89.3% capacity after 100 cycles at − 20 °C and 99.1% at 60 °C, demonstrating strong low-temperature adaptability and excellent high-temperature stability. The Li||NCM811 pouch cells cycled stably at 4.5 V for 30 cycles, and NCM811||graphite pouch cells retained 93.4% of their capacity after 1100 cycles at 1 C. These results demonstrate the effectiveness of electrolyte engineering in enhancing the performance of LMBs and underscore the potential of our approach to advance high-energy and high-power density battery technologies.

## Supplementary Information

Below is the link to the electronic supplementary material.

Supplementary file1 (DOCX 5387 kb)
