# Supplementary material for: Electron Acceptor-Driven Solid Electrolyte Interphases with Elevated LiF Content for 4.7 V Lithium Metal Batteries
Source: Nanomicro Lett. 2025 Feb 24;17:163. doi: 10.1007/s40820-025-01663-x (PMC11850700; doi:10.1007/s40820-025-01663-x)
Supplement: Supplementary file 1 — Supplementary file1 (DOCX 5387 kb) [file 40820_2025_1663_MOESM1_ESM.docx]

Supporting Information for

**Electron Acceptor-Driven Solid Electrolyte Interphases with Elevated LiF Content for 4.7 V Lithium Metal Batteries**

Yongbiao Mu^1,2^‡, Zifan Liao^1,2^‡, Youqi Chu^1,2^‡, Qing Zhang^1,2^‡, Lingfeng Zou^1,2^, Lin Yang^1,2^, Yitian Feng^1,2^, Haixiang Ren^1,2^, Meisheng Han ^1,2^, Lin Zeng ^1,2^*

^1^Shenzhen Key Laboratory of Advanced Energy Storage, Department of Mechanical and Energy Engineering, Southern University of Science and Technology, Shenzhen 518055, P. R. China

^2^SUSTech Energy Institute for Carbon Neutrality, Southern University of Science and Technology, Shenzhen 518055, P. R. China

‡ Yongbiao Mu, Zifan Liao, Youqi Chu, and Qing Zhang contributed equally to this work.

*Corresponding author. E-mail: [zengl3@sustech.edu.cn](mailto:zengl3@sustech.edu.cn) (Lin Zeng)

**S1 Electrochemical Measurements**

The ionic conductivity of the separator immersed in the electrolyte is calculated using the formula: σ_s_ = d/(A×R_s_), where σ_s_ represents the conductivity of the wetted separator, d is the thickness of the separator, A is the contact area, and R_s_ is the impedance of the stainless steel|electrolyte-wetted separator|stainless steel symmetric cell, determined from the x-axis intercept of the Nyquist plot.

The lithium-ion transference number is typically measured using the Bruce-Vincent (BV) method. First, a symmetric Li||Li cell is assembled with an electrolyte. A small constant voltage (10 mV) is applied, and the initial current (I₀) and the steady-state current (Iₛ) after polarization are recorded. Electrochemical impedance spectroscopy is performed both before and after polarization to obtain the impedance values (R₀ and Rₛ). The lithium-ion transference number is then calculated using the formula$t^{+}=\frac{I_{s}*(V-I_{0}*R_{0})}{I_{0}*(V-I_{s}*R_{s})}$, where V is the applied voltage.

For SEM, XPS, and TEM characterization, Li||Cu cells were assembled, and Li plating was carried out for 1 h at a current density of 1.0 mA cm⁻².

The electrochemical performance was evaluated using a Neware battery test system (Shenzhen, China) at 30 °C. Cyclic voltammetry (CV) curves at different scan rates and electrochemical impedance spectroscopy (EIS) measurements from 10⁵ to 10⁻² Hz with an amplitude of 5 mV were obtained using an electrochemical workstation (1470E, Solartron Analytical, UK). The rate performance and cycling performance of the Li||NCM811 and Li||LNMO batteries were tested using the Neware battery testing system.

**S2 *In-situ* XRD Characterization**

The *in-situ* XRD observation cells were assembled using molds obtained from Beijing Scistar Technology Co., Ltd. The working electrode was NCM811, while the counter electrode was lithium metal. The electrolyte used was a custom formulation consisting of 1.5% TPFPB and 6% LiNO_3_ in EMC/FEC. Charging and discharging tests were conducted using a Neware battery test system, with a rate of 0.2C applied to the Li||NCM811 cells. X-ray diffraction (XRD) patterns were acquired using a Bruker Advance D8 Ultima IV equipped with the appropriate instrumentation.

***S3 In-situ* Optical Microscope Characterization**

The cells for *in situ* optical microscopy observation were assembled using molds from Beijing Scistar Technology Co., Ltd. Both the working and counter electrodes were lithium metal. The electrolyte consisted of 1.5% TPFPB and 6% LiNO_3_ in EMC/FEC. An electrochemical workstation was used to supply power, applying a current density of 10 mA/cm².

**S4 FIB-SEM Characterization**

To investigate the structural evolution of cathode material particles after reaction under high voltage, we conducted a detailed analysis of the post-reaction NCM811 cathode material using a TESCAN AMBER FIB-SEM system. First, ion beam milling (30kV, 20nA) was used to precisely cut the sample and obtain cross-sectional views. To remove surface damage caused by milling, a coarse polishing process (30kV, 2.5nA) was applied. Next, high-resolution SEM imaging (20kV, 300pA) was performed to observe the morphology, structural integrity, and possible crack distribution within the particles. Additionally, energy dispersive spectroscopy (EDS) (20kV, 1nA) was employed to analyze the elemental composition of the sample, providing qualitative and quantitative data on the material's composition.

**S5 Molecular Dynamics Simulations**

Molecular dynamics (MD) simulations were conducted using Gromacs (version 2019.5, http://www.gromacs.org). For the FEC/EMC electrolyte, the simulation box (8 × 8 × 8 nm) was filled with 348 FEC, 566 EMC, and 83 LiPF_6_ molecules, adhering to experimental density and stoichiometry. For the electrolyte with TFPFB additive, the box contained 348 EC, 566 EMC, 3 TFPFB, and 83 LiPF_6_ molecules. The OPLS-AA force field was employed to describe inter- and intra-molecular interactions, with parameters generated automatically using the LigParGen Server. Force field parameters were provided in .itp files, including nonbonding parameters for solvent molecules.

Simulations began with a 2 ns NPT run at 500 K, followed by a 3 ns NPT annealing process, cooling the system from 330 K to room temperature (298 K). This prepared a homogeneous single-phase solution as the initial configuration. The system was then equilibrated with a 5 ns NPT and a 10 ns NVT simulation before conducting a 5 ns production run for radial distribution function (RDF) calculations. Temperature and pressure were controlled using the Nose-Hoover thermostat and Berendsen barostat, respectively, with a time step of 1.0 fs. MD trajectory snapshots were visualized using VMD.

**S6 Quantum-Chemical Calculations**

Quantum-chemical calculations were performed to determine the highest occupied molecular orbital (HOMO) and lowest unoccupied molecular orbital (LUMO) energies, Fukui function, and electrostatic potential (ESP). Structures were optimized using the B3LYP method with the 6-311G+ (d, p) basis set, and energy calculations were performed with the same method and basis set. Binding energy was calculated using Gaussian 16 with the formula:

$$\Delta G_{binding}= E_{AB}-E_{A}-E_{B}$$

where $E_{AB}$is the Gibbs free energy of the AB complex, $E_{A}$ is the Gibbs free energy of component A, and $E_{B}$is the Gibbs free energy of component B. Desolvation energy was calculated similarly.

**S7 Adsorption Energy Calculations**

Adsorption energy calculations were carried out using the Vienna Ab-initio Simulation Package (VASP), based on density functional theory. Core and valence electron interactions were described using projected augmented wave (PAW) methods, with local density approximated by the generalized gradient approximation (GGA) with PBE exchange-correlation energy. Brillouin zone sampling was performed using the Monkhorst-Pack method, with a cutoff energy of 400 eV. K-points ranging from 1×1×1 to 3×3×1 were used to optimize convergence in diffusion barrier and mechanical strength calculations. Convergence criteria for electron and ion relaxation were set to 1.0 × 10⁻⁴ eV and 1.0 × 10⁻³ eV, respectively, and force convergence was set at 0.02 eV/Å. Decomposition energies (E_de) were calculated using:

$$E_{b}= E_{de/sub}-E_{FEC}-E_{sub}$$

where $E_{de/sub}$ is the total energy of the optimized decompose/substrate system, $E_{FEC}$ is the energy of FEC in the structure, and $E_{sub}$ is the energy of the clean substrate.

**S8 Reporting Summary**

Further information on research design is available in the [Nature Portfolio Reporting Summary](https://www.nature.com/articles/s41467-024-52234-4#MOESM3) linked to this article.

**Supplementary Figures and Tables**


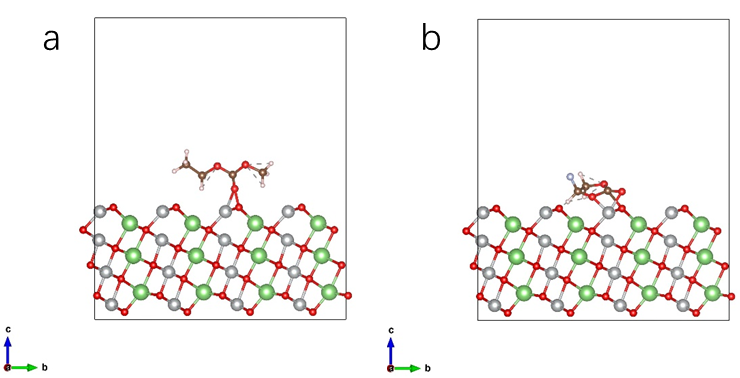


**Fig. S1** DFT calculation of H adsorption energy of **a**) EMC and **b**) FEC on the surface of NCM811


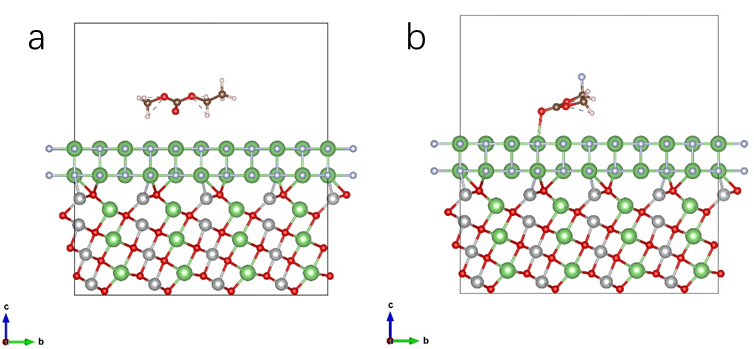


**Fig. S2** DFT calculation of adsorption energy of **a**) EMC and **b**) FEC on the surface of the NCM811/LiF layer.


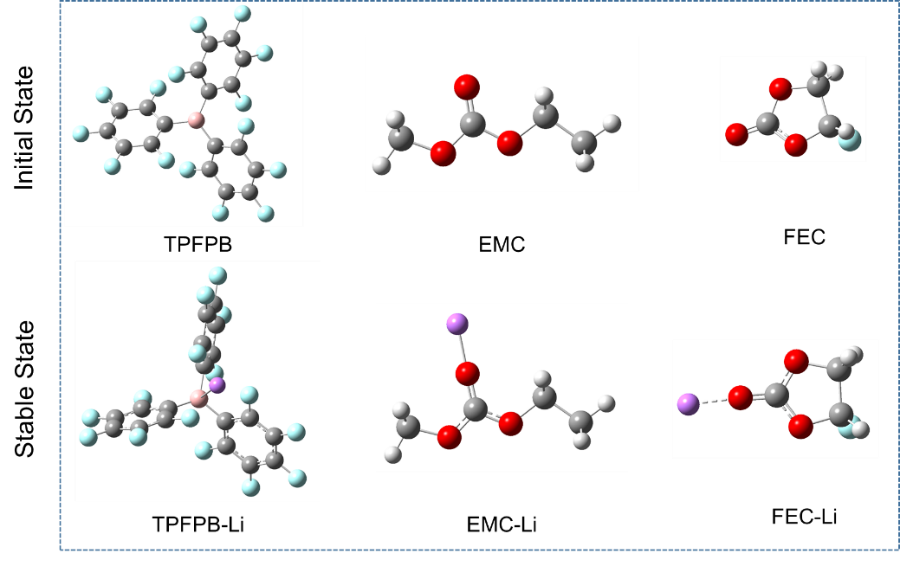


**Fig. S3** DFT calculation of lithium-ion binding energy calculation: molecular structures before and after geometry optimization


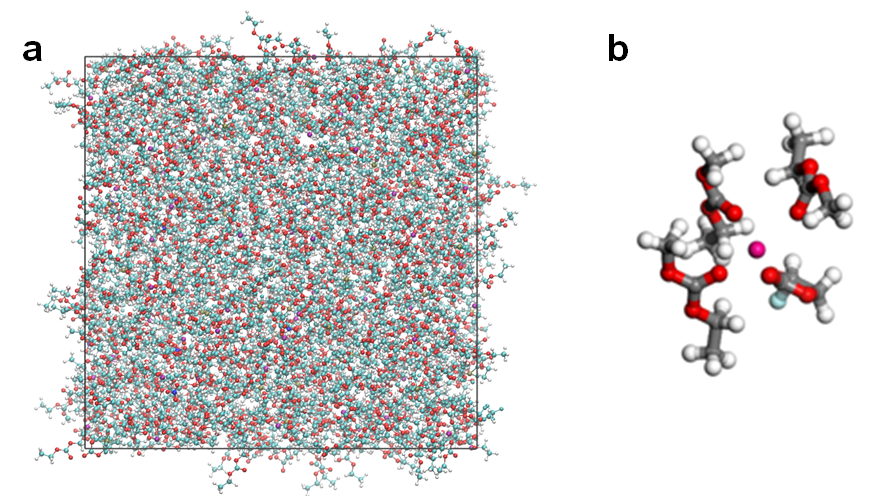


**Fig. S4** MD simulation of the electrolyte system without TPFPB and its enlarged structure


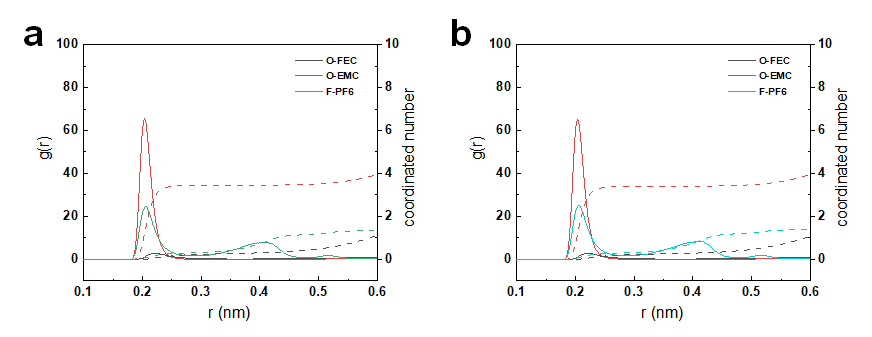


**Fig. S5** Radial distribution function and coordination number of O-FEC, O-EMC, and F-PF_6_ in **a**) additive-free electrolyte and **b**) TPFPB-modified electrolyte


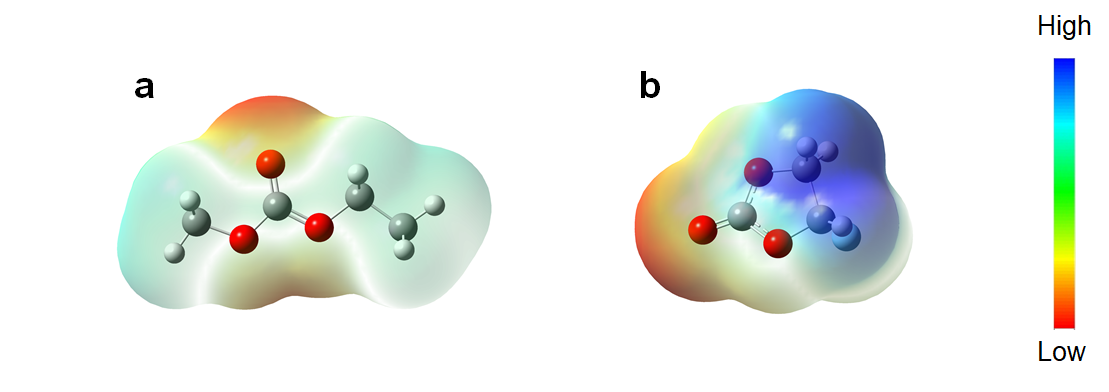


**Fig. S6** Electrostatic potential density distribution of **a**) EMC and **b**) FEC molecules


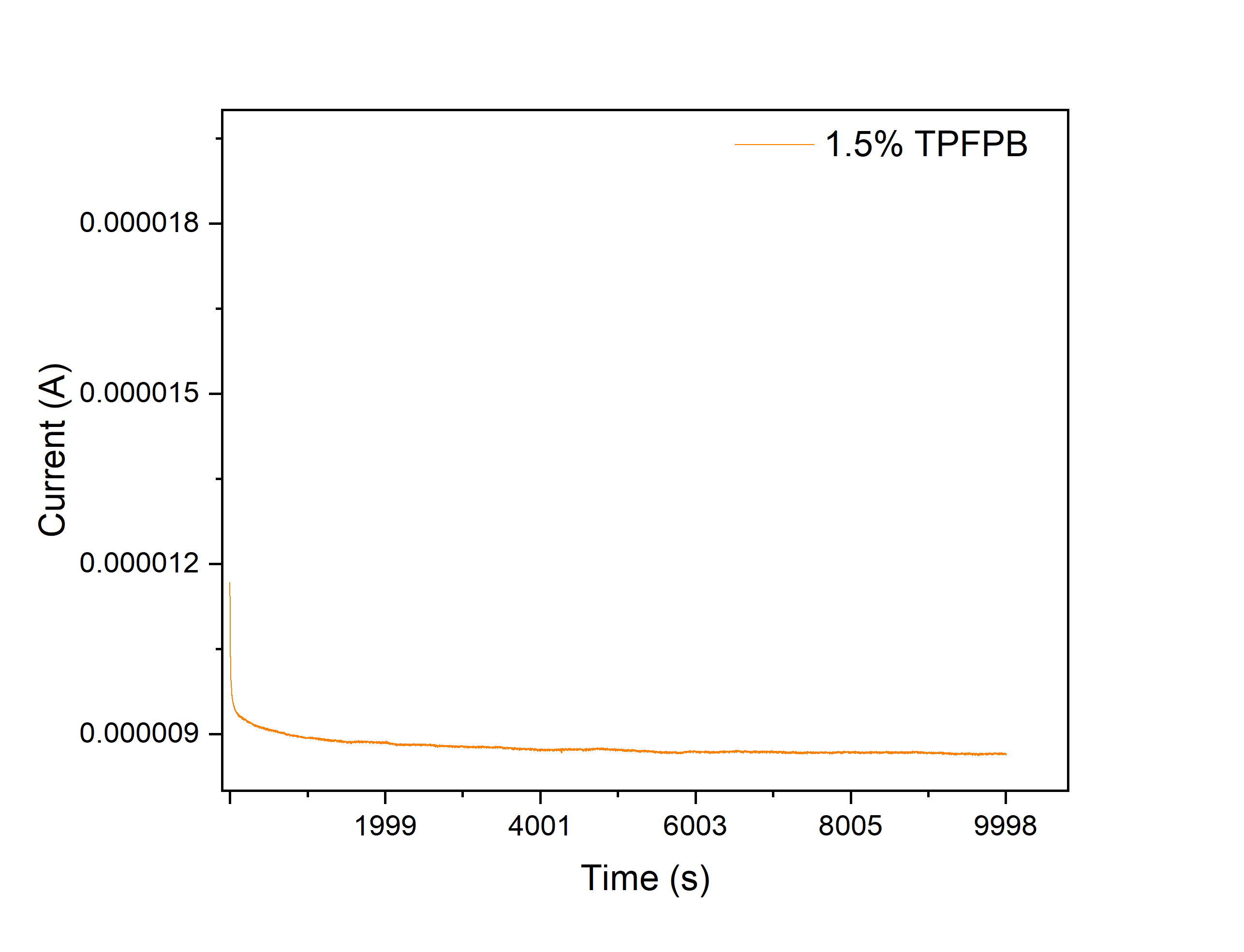


**Fig. S7** The i-t curve for Li||Li cells with 1.5% TPFPB-modified electrolye


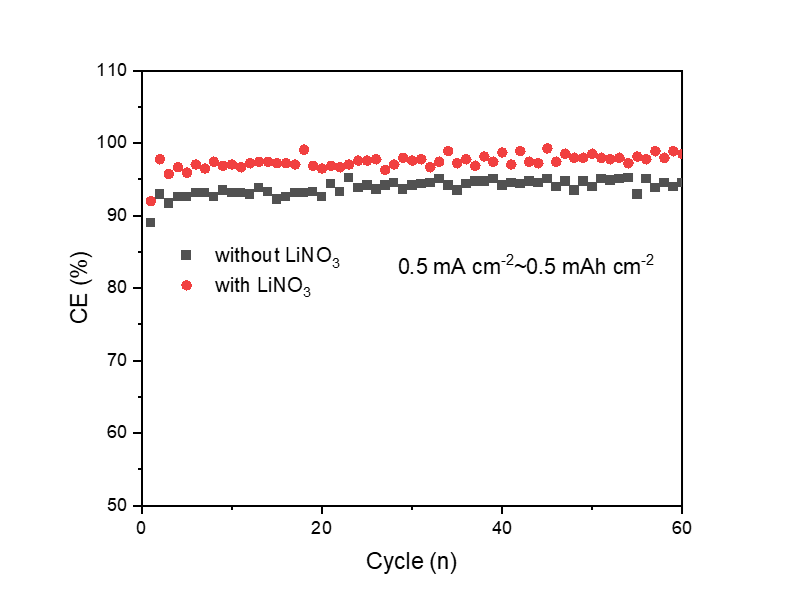


**Fig. S8** CE of Li|Cu batteries at 0.5mA cm^-2^ and 0.5 mAh cm^-2^


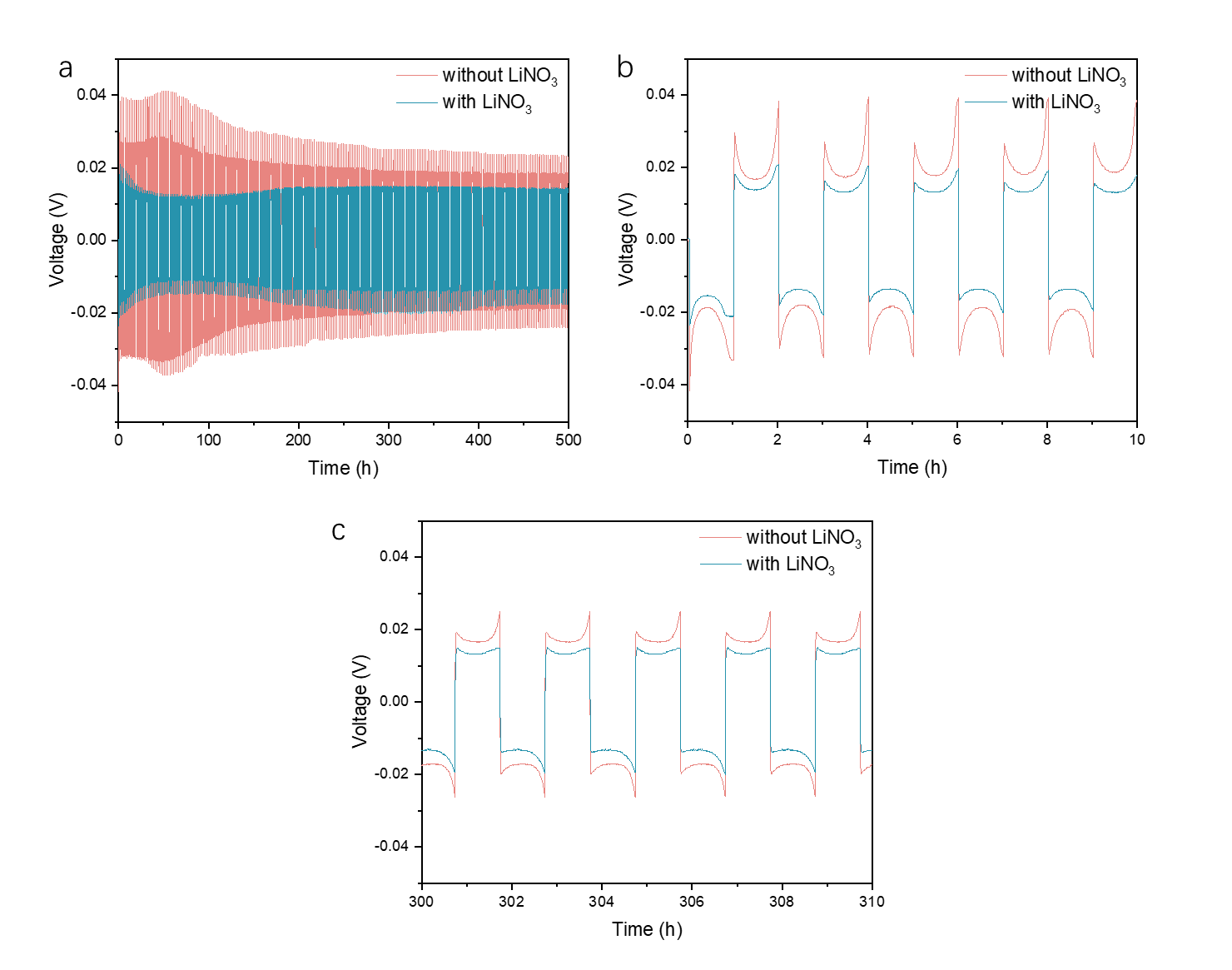


**Fig. S9 a**) Cycling stability of Li|Li batteries at 0.25mA cm^-2^ and 0.25 mAh cm^-2^; **b, c**) the corresponding enlarged plating and stripping curves during different time


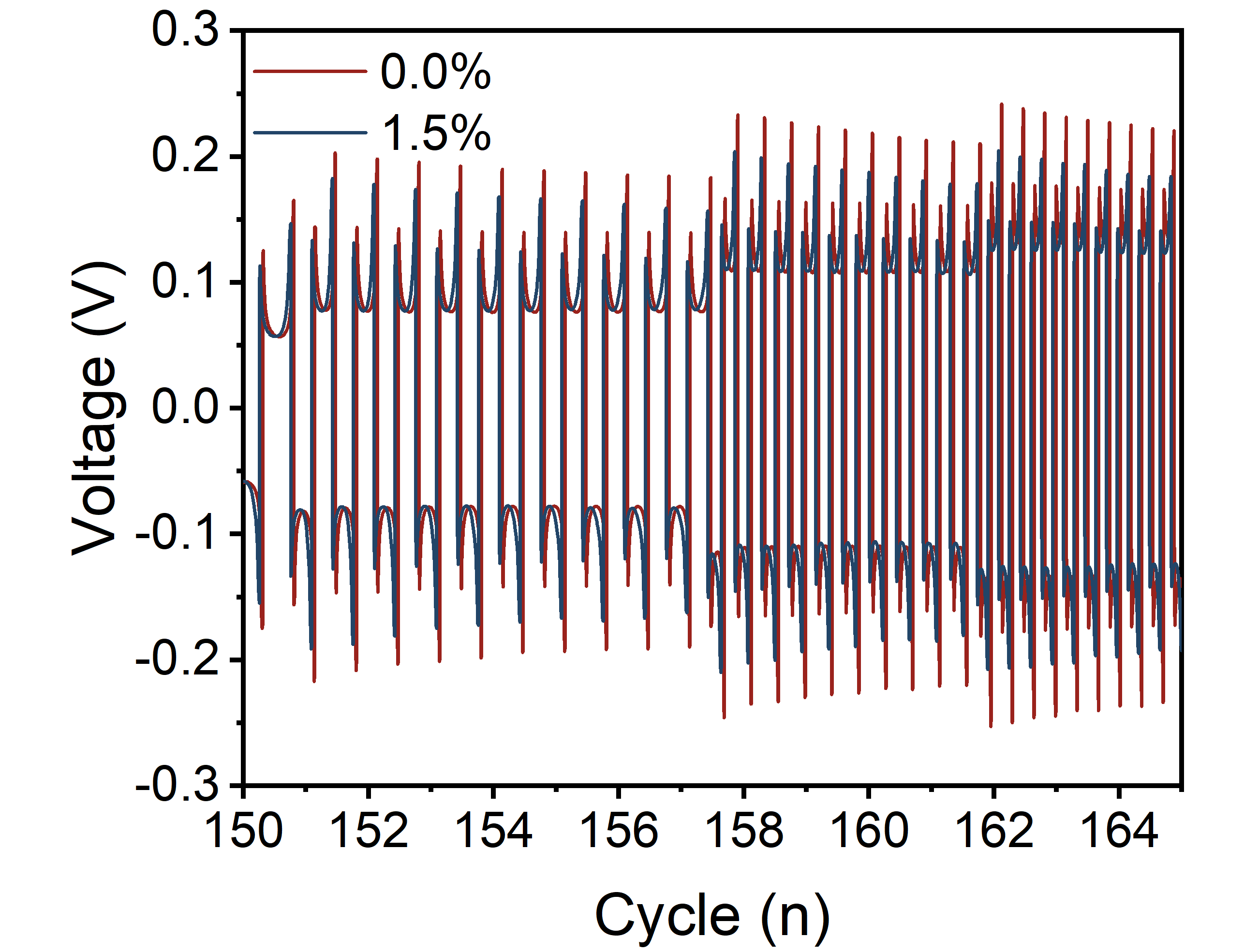


**Fig. S10** Rate performance of Li||Li symmetric cells under the current densities of 6 mA cm^-2^, 8 mA cm^-2^, and 10 mA cm^-2^


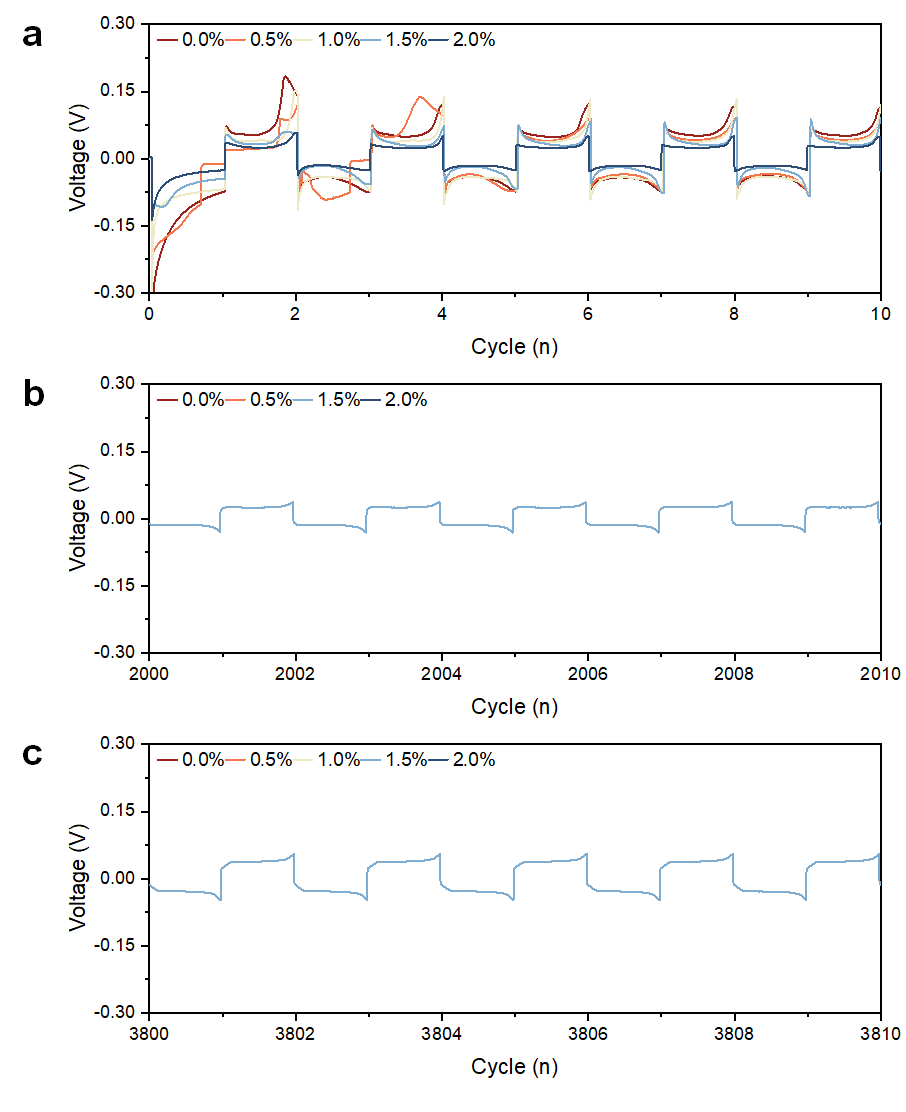


**Fig. S11** The enlarged plating and stripping curves for Li||Li symmetric cells at different stages: **a**) 0-10h, **b**) 2000-2010h, and **c**) 3800-3810h


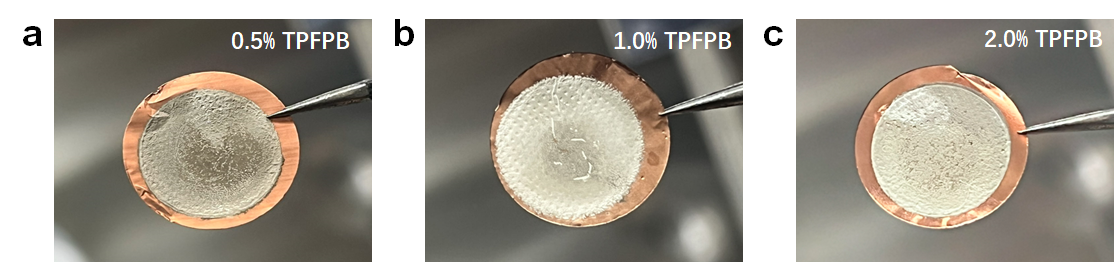


**Fig. S12** Optical images of 1 mAh Li metal deposition **a**) 0.5% TPFPB additive, **b**) 1.0% TPFPB additive and **c**) 2.0% TPFPB additive


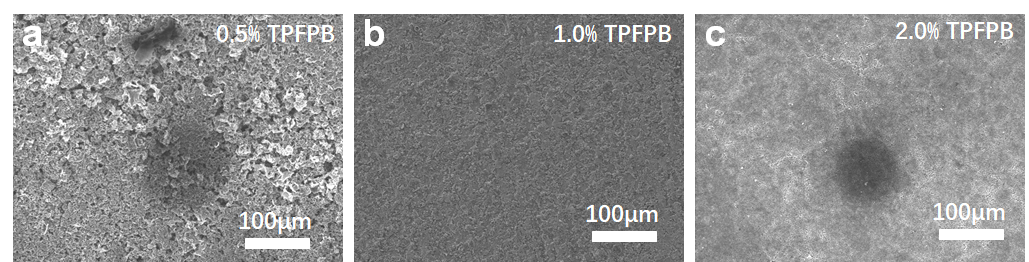


**Fig. S13** SEM images of 1 mAh Li metal deposition **a**) 0.5% TPFPB additive, **b**) 1.0% TPFPB additive and **c**) 2.0% TPFPB additive.


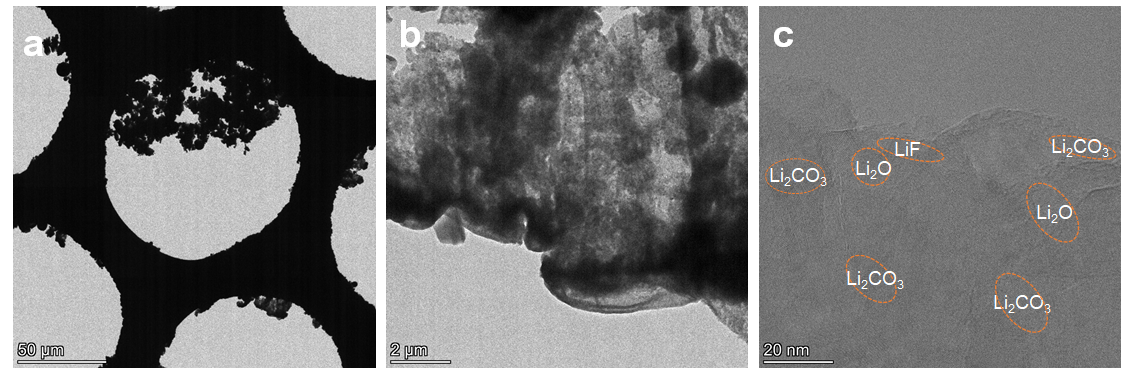


**Fig. S14** Cryo-TEM images of the SEI formed at 30°C for the deposited Li with additive-free electrolyte: **a**) Li/Cu mesh image, **b**) low-resolution TEM, and **c**) High-resolutionTEM images of the SEI.


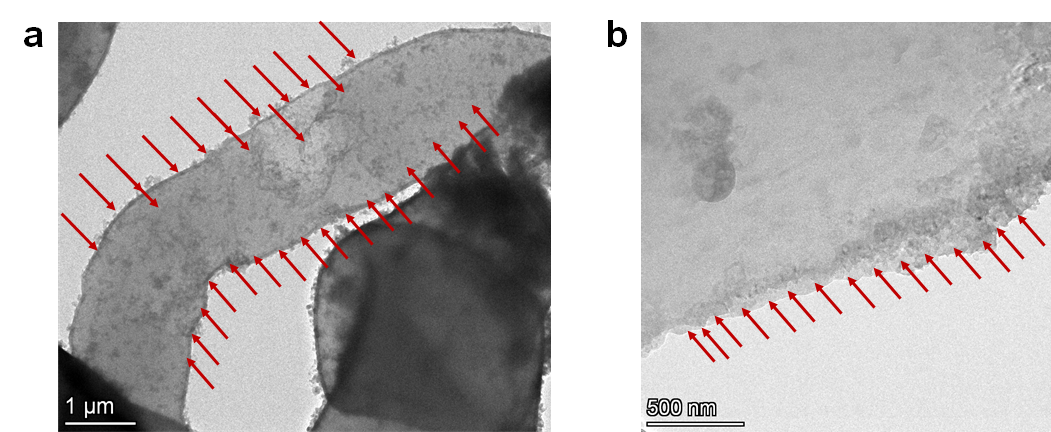


**Fig. S15** Cryo-TEM images of the SEI formed at 30°C for the deposited Li with TPFPB-modified electrolyte: **a**) low-resolution TEM, and **b**) High-resolutionTEM images of the SEI


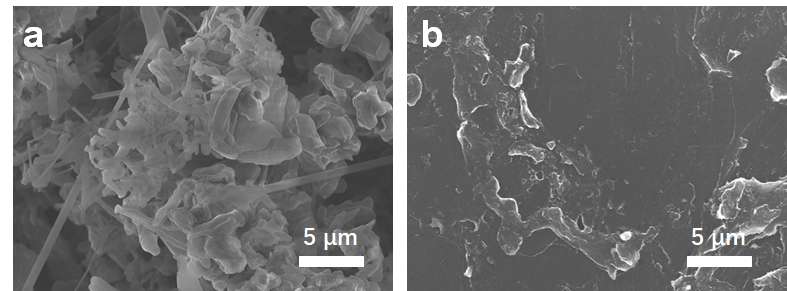


**Fig. S16** TEM images of deposited Li in **a**) the blank electrolyte and **b**) TPFPB-modified electrolyte


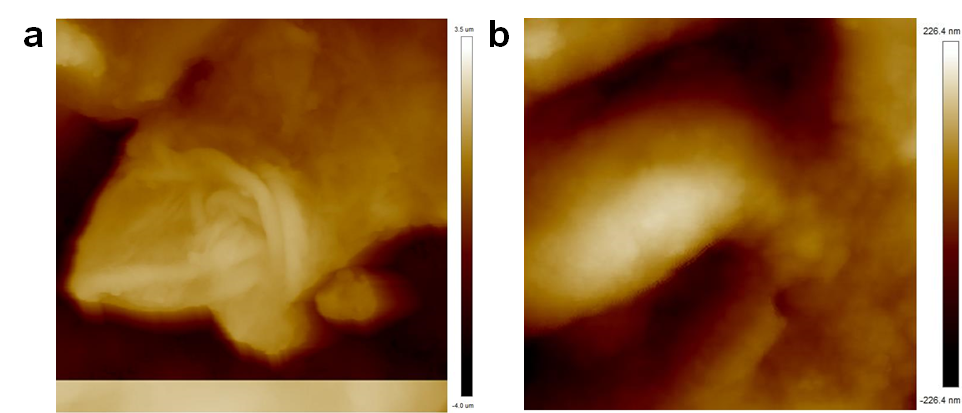


**Fig. S17** AFM images of deposited Li in a) the blank electrolyte and b) TPFPB-modified electrolyte


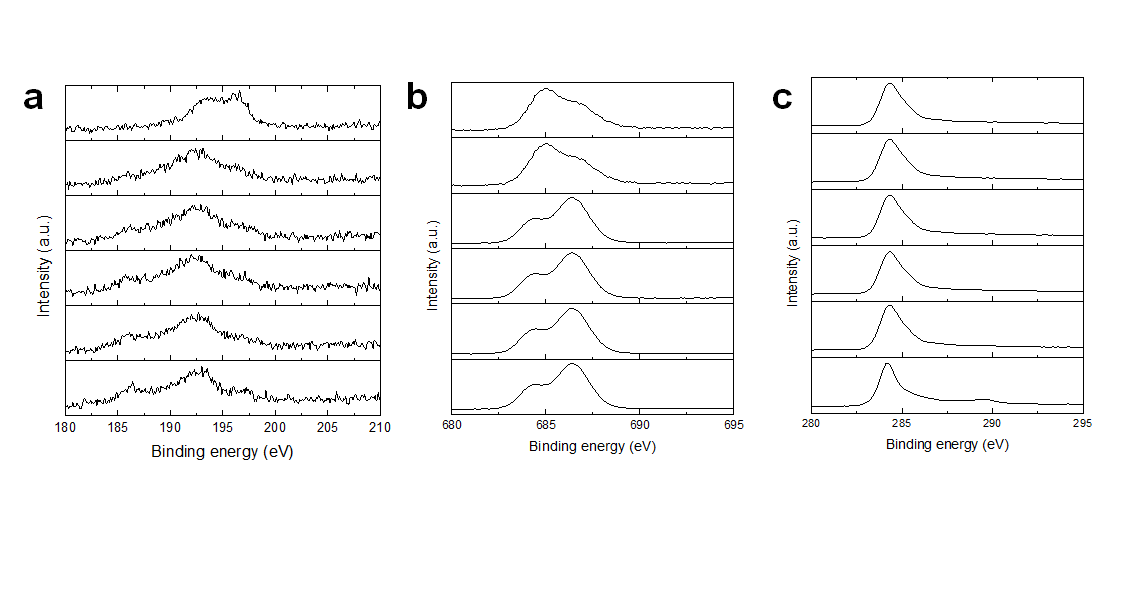
**Fig. S18** XPS spectra of **a**) B *1s*, **b**) F *1s*, and **c**) C *1s* for the SEI formed on deposited Li after 5 cycles in Li||Cu asymmetrical cells with modified electrolytes


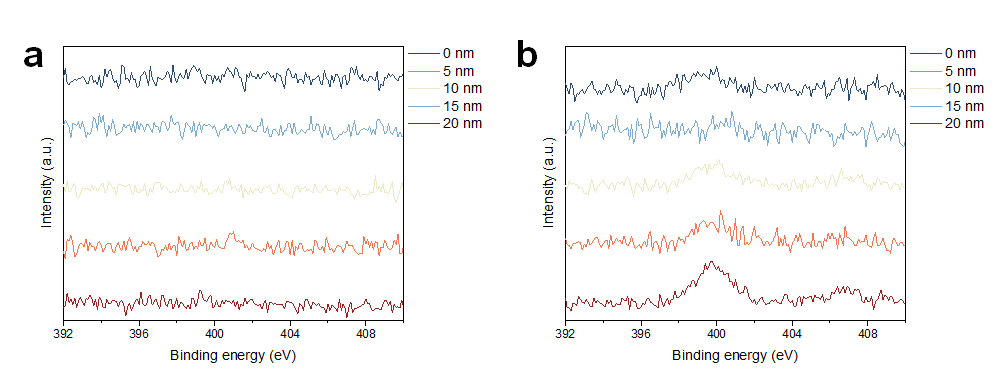
**Fig. S19** XPS spectra of N *1s*for the SEI formed on deposited Li after 5 cycles in Li||Cu asymmetrical cells with **a**) additive-free and **b**) TPFPB-modified electrolytes


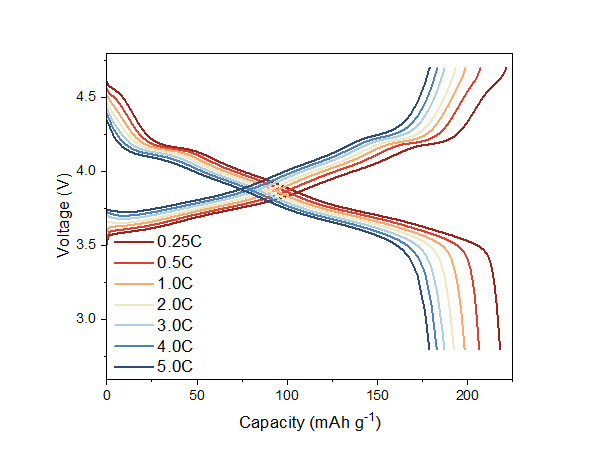


**Fig. S20** The corresponding charge-discharge curves of Li||NCM811 cells with TPFPB-modified electrolyte at 4.7 V and under different rates


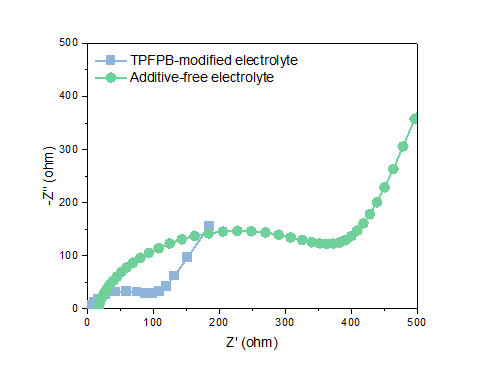


**Fig. S21** EIS curves of Li||NCM811 cells with additive-free and TPFPB-modified electrolytes

**Fig. S22** Cycling stability of Li|NCM811 batteries at **a**) -20 °C and **b**) 60 °C

**Fig. S23** Cycling stability of Li|LNMO batteries at **a**) -20 °C and **b**) 60 °C


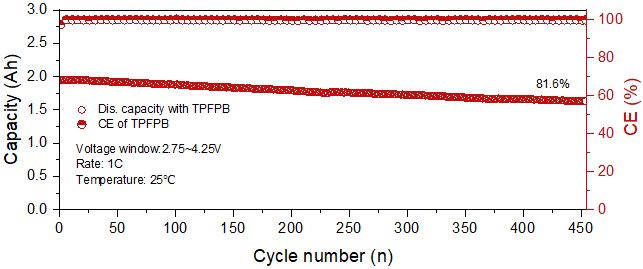


**Fig. S24** Cycling stability of graphite||NCM811 pouch cell at 1.0C with additive-free electrolytes


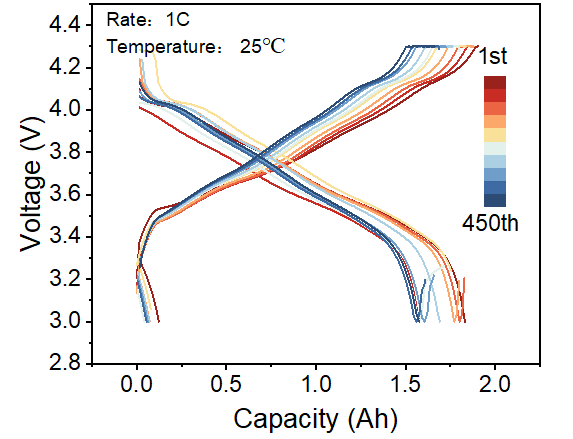


**Fig. S25** The corresponding charge and discharge curves of graphite||NCM811 pouch cell at 1.0C with additive-free electrolytes


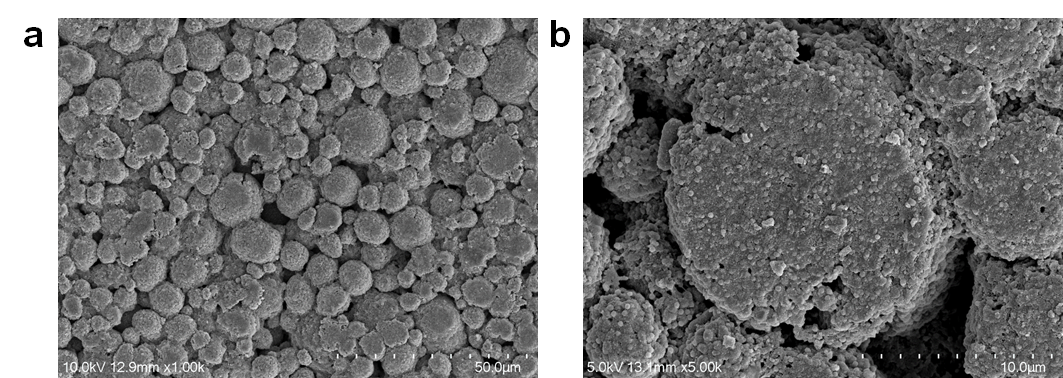


**Fig. S26** SEM images of the NCM811 electrode after 100 cycles with additive-free electrolyte under 4.7V


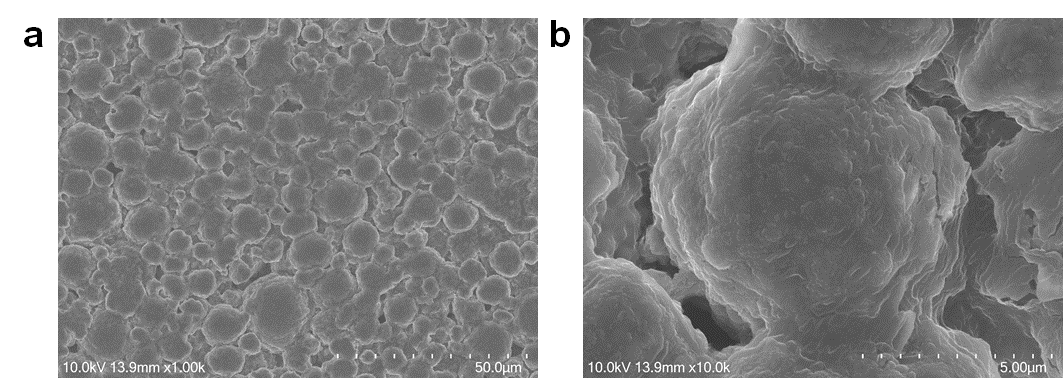


**Fig. S27** SEM images of the NCM811 electrode after 100 cycles with TPFPB-modified electrolyte under 4.7V


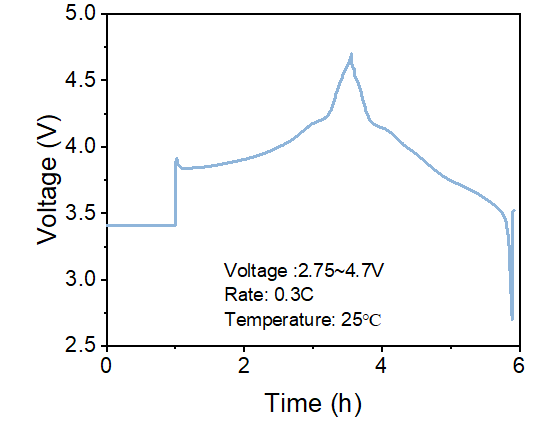


**Fig. S28** The charge-discharge curve of the Li||NCM811 cell with TPFPB-modified electrolyte under 3.75~4.7 V during the *in-situ* XRD test


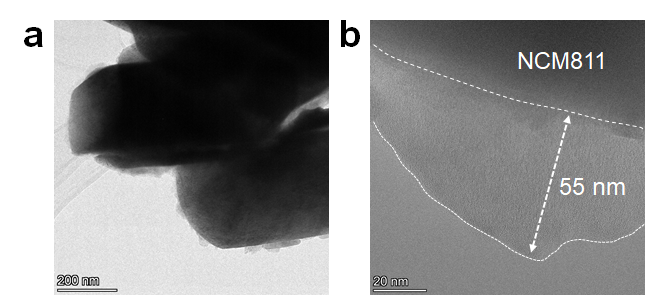


**Fig. S29** Cryo-TEM images of the CEI film formed at 30°C: **a**) Low magnification cryo-TEM images; **b**) High-resolution cryo-TEM image of the CEI formed in additive-free electrolyte

**Table S1** The ionic conductivity of electrolytes with different TPFPB amount

| TPFPB (%) | Ionic conductivity (mS/cm) |
| --- | --- |
| 0 | 0.35 |
| 0.5 | 0.39 |
| 1.0 | 0.51 |
| 1.5 | 0.68 |
| 2.0 | 0.61 |

**Table S2** Li⁺ ion transference number with different TPFPB concentration

| TPFPB (%) | Li⁺ ion transference number |
| --- | --- |
| 0 | 0.41 |
| 0.5 | 0.52 |
| 1.0 | 0.61 |
| 1.5 | 0.71 |
| 2.0 | 0.59 |

**Table S3** Electrochemical window of the electrolytes with different TPFPB concentration

| TPFPB (%) | Electrochemical window (V) |
| --- | --- |
| 0 | 4.7 |
| 0.5 | 4.91 |
| 1.0 | 5.03 |
| 1.5 | 5.41 |
| 2.0 | 5.18 |

**Table S4** The average coulombic efficiency (a-CE) and over-potential of Li||Cu cells using the electrolytes with different TPFPB concentration

| TPFPB (%) | a-CE (% | Over-potential (mV) |
| --- | --- | --- |
| 0 | 101.05 | 57.6 |
| 0.5 | 95.50 | 39.3 |
| 1.0 | 96.78 | 37.5 |
| 1.5 | 98.51 | 35.3 |
| 2.0 | 97.20 | 38.5 |

**Table S5** The discharge capacity of Li||NCM811 cells with different electrolytes under different rates

| Electrolytes | 0.25C | 0.5C | 1.0C | 2.0C | 3.0C | 4.0C | 5.0C |
| --- | --- | --- | --- | --- | --- | --- | --- |
| Additive-free | 203.9 | 198.4 | 190.6 | 181.9 | 176.2 | 169.2 | 157.8 |
| TPFPB-modified | 226.8 | 218.0 | 207.4 | 196.7 | 189.4 | 182.0 | 168.3 |

**Table S6** The discharge capacity of Li||LNMO cells with different electrolytes under different rates

| Electrolytes | 0.25C | 0.5C | 1.0C | 2.0C | 5.0C | 10.0C |
| --- | --- | --- | --- | --- | --- | --- |
| Additive-free | 137.9 | 137.5 | 136.6 | 134.6 | 134.4 | 132.3 |
| TPFPB-modified | 125.8 | 113.3 | 96.7 | 76.7 | 66.7 | 52.5 |

**Table S7** Comparison of pouch cells with NCM811/Graphite reported in the literature

| Electrolytes | Capacity (Ah) | Rate (C) | Cycles | Retention (%) | Temperature (℃) | Refs. |
| --- | --- | --- | --- | --- | --- | --- |
| C=C/C≡C/Siloxane | 1.0 | 0.5 | 265 | 85 | 60 | [S1] |
| CTFP | 1.8 | 1.0 | 200 | 75 | 25 | [S2] |
| PDTD | 1.0 | 1.0 | 900 | 85 | 55 | [S3] |
| PhIm-TfO | 1.2 | 1.0 | 500 | 85 | 45 | [S4] |
| HFB | 1.5 | 1.0 | 1150 | 91 | 25 | [S5] |
| TFA/FEC | 0.73 | 1.0 | 400 | 82 | 45 | [S6] |
| TPBX | 1.0 | 0.5 | 750 | 66 | 25 | [S7] |
| WNLE | 0.8 | 3.0 | 700 | 82 | 45 | [S8] |
| DTDS | 1.0 | 0.5 | 610 | 91.4 | 25 | [S9] |
| FEMC | 1.2 | 1.0 | 1200 | 75.6 | / | [S10] |
| LiDOP | 3.0 | 0.5 | 150 | 85.6 | / | [S11] |
| mFT-LHCE | 1.2 | 0.5 | 130 | 90.4 | 25 | [S12] |
| TPFPB/LiNO_3_ | 2.0 | 1.0 | 1160 | 93.4 | 25 | This work |

**Supplementary References**

1. Y. Chen, Q. He, Y. Mo, W. Zhou, Y. Zhao et al., Engineering an insoluble cathode electrolyte interphase enabling high performance NCM811// graphite pouch cell at 60 ℃. Adv. Energy Mater. **12**, 2201631 (2022). <https://doi.org/10.1002/aenm.202201631>
2. X. Li, Z. Xie, K. Zhou, J. Li, X. Zhang et al., Constructing a stable interface film on both cathode and anode *via* a novel electrolyte additive for high performance LiNi_0.8_Co_0.1_Mn_0.1_O_2_/graphite pouch cell. J. Power Sources **596**, 234055 (2024). <https://doi.org/10.1016/j.jpowsour.2024.234055>
3. H. Zhao, S. Hu, Y. Fan, Q. Wang, J. Li et al., Significance of electrolyte additive molecule structure in stabilizing interphase in LiNi_0.8_Co_0.1_Mn_0.1_O_2_/artificial graphite pouch cells at high temperature. Energy Storage Mater. **65**, 103151 (2024). <https://doi.org/10.1016/j.ensm.2023.103151>
4. W. Kim, T.H. Kim, J. Yu, Y.-J. Kim, K.J. Kim et al., Interface-targeting individually functionalized ionic additive to construct stable interphase on selective electrode surface for practical lithium-ion pouch cells. Adv. Funct. Mater. **33**, 2306068(2023). <https://doi.org/10.1002/adfm.202306068>
5. M. Yang, K. Chen, H. Li, Y. Cao, H. Yang et al., Molecular adsorption-induced interfacial solvation regulation to stabilize graphite anode in ethylene carbonate-free electrolytes. Adv. Funct. Mater. **33**, 2306828 (2023). <https://doi.org/10.1002/adfm.202306828>
6. K. An, Y.H.T. Tran, S. Kwak, J. Han, S.-W. Song, Design of fire-resistant liquid electrolyte formulation for safe and long-cycled lithium-ion batteries. Adv. Funct. Mater. **31**, 2106102 (2021). <https://doi.org/10.1002/adfm.202106102>
7. W. Li, A. Zhu, H. Gu, B. Wang, G. Wang et al., Co-passivation of perovskite film towards stable and efficient perovskite solar cell. Chem. Eng. J. **471**, 144561 (2023). <https://doi.org/10.1016/j.cej.2023.144561>
8. K. An, D. Kim, Y.H.T. Tran, D.T.T. Vu, S.J. Park et al., Weakly binding molecules-based fast charging Li-ion batteries. Adv. Funct. Mater. **34**, 2311782 (2024). <https://doi.org/10.1002/adfm.202311782>
9. Z. Ru, M. Peng, S. Amzil, S. Luo, T. Xu et al., A synergistic Duo for enhanced cathode stability in high-voltage lithium-ion batteries. Energy Storage Mater. **70**, 103537 (2024). <https://doi.org/10.1016/j.ensm.2024.103537>
10. M. Qin, Z. Zeng, F. Ma, C. Gu, X. Chen et al., Doping in solvation structure: enabling fluorinated carbonate electrolyte for high-voltage and high-safety lithium-ion batteries. ACS Energy Lett. **9**, 2536–2544 (2024). <https://doi.org/10.1021/acsenergylett.4c00790>
11. X. Wu, Y. Xue, Z. Li, M. Huang, X. Song et al., Molecular design of highly Li-ion conductive cathode-electrolyte interface enabling excellent rate performance for lithium-ion batteries. Chem. Eng. J. **493**, 152512 (2024). <https://doi.org/10.1016/j.cej.2024.152512>
12. M. Fang, B. Du, X. Zhang, X. Dong, X. Yue et al., An electrolyte with less space-occupying diluent at cathode inner Helmholtz plane for stable 4.6 V lithium-ion batteries. Angew. Chem. Int. Ed. **63**, e202316839 (2024). <https://doi.org/10.1002/anie.202316839>
